# Origin and Acceleration of Insoluble Li_2_S_2_−Li_2_S Reduction Catalysis in Ferromagnetic Atoms‐based Lithium‐Sulfur Battery Cathodes

**DOI:** 10.1002/anie.202215414

**Published:** 2022-12-01

**Authors:** Rui Yan, Zhenyang Zhao, Menghao Cheng, Zhao Yang, Chong Cheng, Xikui Liu, Bo Yin, Shuang Li

**Affiliations:** ^1^ College of Polymer Science and Engineering State Key Laboratory of Polymer Materials Engineering Sichuan University Chengdu 610065 China; ^2^ Department of Chemistry Technische Universität Berlin Berlin 10623 Germany

**Keywords:** Energy Storage, Ferromagnetic Element, Insoluble Li_2_S_2_−Li_2_S Reduction, Lithium-Sulfur Battery, Polar Single-Atom Catalysts

## Abstract

Accelerating insoluble Li_2_S_2_−Li_2_S reduction catalysis to mitigate the shuttle effect has emerged as an innovative paradigm for high‐efficient lithium‐sulfur battery cathodes, such as single‐atom catalysts by offering high‐density active sites to realize in situ reaction with solid Li_2_S_2_. However, the profound origin of diverse single‐atom species on solid‐solid sulfur reduction catalysis and modulation principles remains ambiguous. Here we disclose the fundamental origin of Li_2_S_2_−Li_2_S reduction catalysis in ferromagnetic elements‐based single‐atom materials to be from their spin density and magnetic moments. The experimental and theoretical studies disclose that the Fe−N_4_‐based cathodes exhibit the fastest deposition kinetics of Li_2_S (226 mAh g^−1^) and the lowest thermodynamic energy barriers (0.56 eV). We believe that the accelerated Li_2_S_2_−Li_2_S reduction catalysis enabled via spin polarization of ferromagnetic atoms provides practical opportunities towards long‐life batteries.

## Graphical abstract

The fundamental origin and acceleration of insoluble Li_2_S_2_−Li_2_S reduction catalysis in ferromagnetic element‐based lithium‐sulfur battery cathodes have been disclosed in this study. We show that the increase of spin polarization degree of metal‐N_4_ sites enhances the metal‐S interaction and accelerates the Li_2_S_2_−Li_2_S reduction catalysis at cathode interface, which provides practical opportunities towards high‐energy and long‐life batteries.

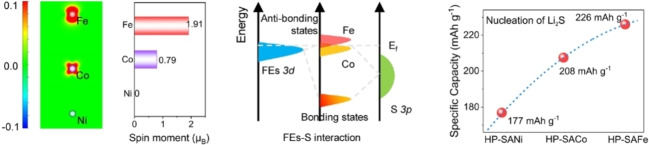

## Introduction

Lithium‐sulfur (Li−S) batteries have been regarded as the most promising energy storage system due to the high theoretical capacity (1675 mAh g^−1^) and natural abundance of sulfur element.^[1]^ The high‐energy and long‐life Li−S batteries rely on the cathodes with efficient polysulfide redox capability. In typical polysulfide redox chemistry, the sulfur reduction reaction (SRR) undergoes a complex conversion process from the sulfur molecule (S_8_) to soluble Li_2_S_*x*_ (LiPSs, 4≤*x*≤8), ultimately generating insoluble Li_2_S_2_ and Li_2_S.^[2]^ Fundamentally, the inherent sluggish SRR kinetics result in low sulfur utilization and shuttle effect of the soluble polysulfides.^[3]^ This has led to two associated trends in recent cathode design of Li−S batteries: catalytic sites with sufficient adsorption/bonding capability to polysulfides and fast catalytic conversion of polysulfide intermediates.

Theoretical calculations suggest that the rate‐determining step in most of the SRR processes is the solid‐solid conversion from Li_2_S_2_ to Li_2_S due to the sluggish solid diffusion and poor interface contact between catalysts and Li_2_S_2_.^[4]^ Therefore, the sluggish electrodeposition of Li_2_S and the associated accumulation of Li_2_S_*x*_ and dead sulfur have long been considered as the root cause for the rapid capacity fading of cathodes.^[5]^ The key point to overcoming this sluggish process relies on a strategy that weakens the S−S bond and promotes the Li_2_S_2_ dissociation to accelerate the insoluble Li_2_S_2_−Li_2_S reduction catalysis.^[6]^ However, the influences and roles of diverse polysulfide redox catalysts on Li_2_S_2_ dissociation remain unclear, which is of great importance to be discovered for the future design of efficient and long‐cycling Li−S battery cathodes.^[7]^

Due to the much less molecular movement ability in the solid phase than that in solution, catalytic sites with high activity and density are needed for the polysulfide redox materials to realize an efficient in situ reaction with solid Li_2_S_2_. Therefore, to accelerate the solid‐solid conversion kinetics, both geometric and electronic structures of the cathode materials should be considered. Single‐atom catalysts (SACs), comprising monodispersed metal active sites offer a theoretical 100 % atom utilization, therefore, will form an atomic‐level contact/catalytic interface for promoting solid‐state Li_2_S_2_/Li_2_S conversion.^[8]^ Recently, diverse “SACs” cathodes have been reported in enhancing Li−S battery performances, for instance, the ferromagnetic elements (Fes=Fe, Co, and Ni)‐based cathodes with metal‐N_4_ structures have been demonstrated to possess enhanced polysulfide catalytic conversion ability.^[9]^ Regrettably, the insoluble Li_2_S_2_−Li_2_S reduction mechanisms via taking the FEs‐based SACs is unclear, and the corresponding correlation between the catalytic activities and electronic structures of FEs−N_4_ remain undiscovered.

In this work, we provide a comparative study on the fundamental origin of insoluble Li_2_S_2_−Li_2_S reduction catalysis in FEs‐based SAC cathodes with different metal‐N_4_ sites. Through a series of theoretical studies, we disclose that the spin polarization (Fe−N_4_>Co−N_4_>Ni−N_4_) can provide spin electrons to reduce antibonding orbitals occupation in Li_2_S_2_−FEs−N_4_ and enhance the FEs−S interaction, thereby weakening the strength of the S−S bond in Li_2_S_2_, and eventually accelerating the Li_2_S_2_−Li_2_S reduction catalysis at cathode interface. Meanwhile, we have synthesized a series of FEs‐based single‐atom sites loaded on hierarchical porous carbon (HP‐SAFEs) as cathode materials to verify the proposed mechanism. Thereafter, systematically spectroscopic, structural, and electrochemical studies have demonstrated that the Fe−N_4_‐based cathodes exhibit the fastest Li_2_S_2_−Li_2_S reduction kinetics and the highest capacity retention of 578 mAh g^−1^ after 200 cycles under 1 C (1 C=1675 mA g^−1^), which is far exceeding those of HP‐SACo (512 mAh g^−1^) and HP‐SANi (454 mAh g^−1^) based batteries. Our findings suggest that the spontaneous spin polarization of ferromagnetic atoms can accelerate insoluble Li_2_S_2_−Li_2_S reduction catalysis, thus offering a new strategy to design high‐energy and long‐life polysulfide reduction catalysts for practical Li−S batteries.

## Results and Discussion

**Figure 1:**
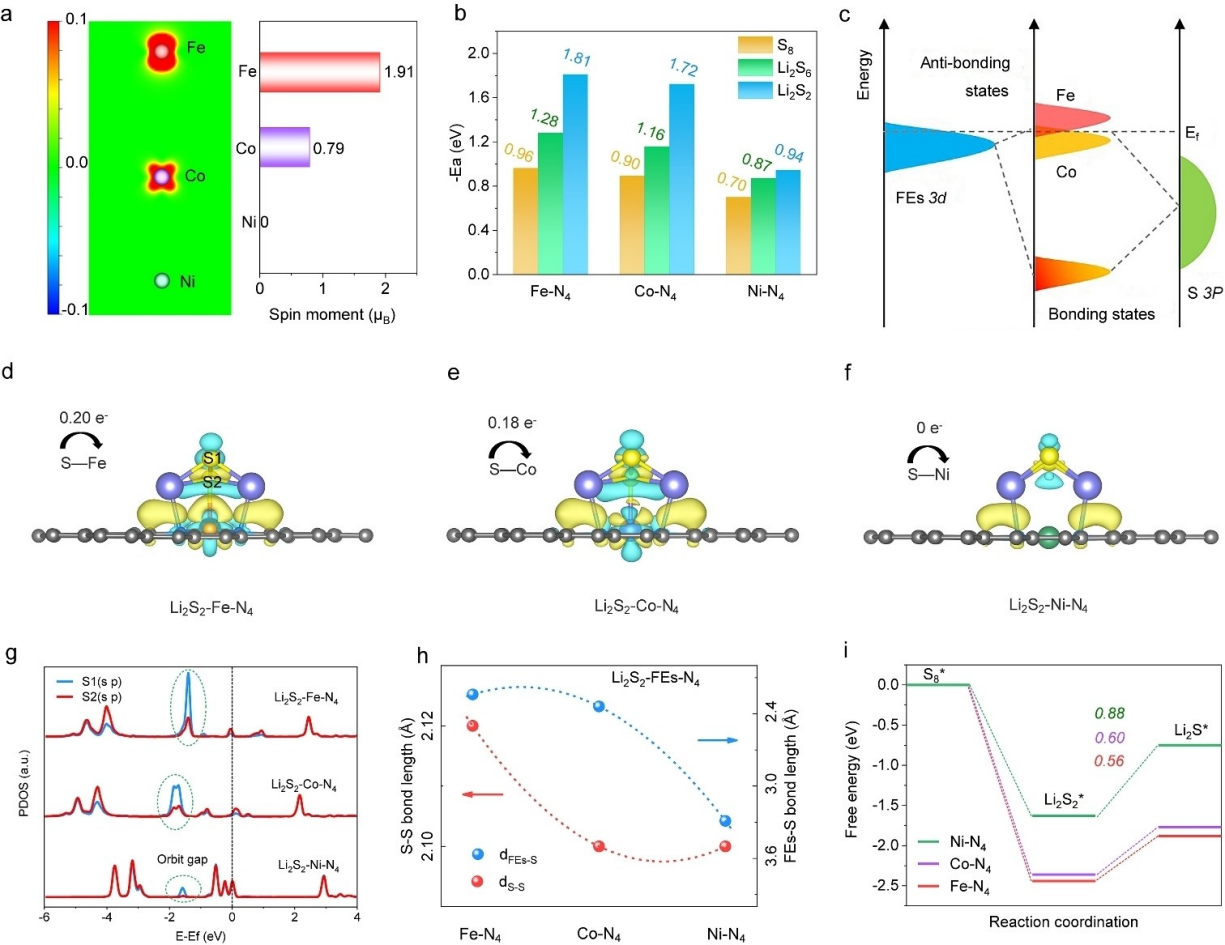
Theoretical understanding for the Li_2_S_2_−Li_2_S reduction catalysis in FEs‐based SAC. a) The calculated spin density and magnetic moment of FEs−N_4_. b) The binding energy of S_8_, Li_2_S_6_, and Li_2_S_2_ with FEs−N_4_. c) Schematic illustration of the local electronic structure of Fe/Co 3*d*‐orbitals in Li_2_S_2_−FEs−N_4_, E_f_ is the Fermi level. The charge density differences of d) Li_2_S_2_−Fe−N_4_, e) Li_2_S_2_−Co−N_4_, and f) Li_2_S_2_−Ni−N_4_. g) The calculated PDOS of S in Li_2_S_2_−Fe−N_4_, Li_2_S_2_−Co−N_4_, and Li_2_S_2_−Ni−N_4_. h) The bond lengths of FEs−S and S−S in Li_2_S_2_−FEs−N_4_, respectively. i) The free energy diagrams for the reduction process of Li_2_S_2_ to Li_2_S on the FEs−N_4_.

To disclose the origin of insoluble Li_2_S_2_−Li_2_S reduction catalysis for the FEs‐based SAC cathodes with different metal‐N_4_ sites, the adsorption and dissociation free energies of Li_2_S_2_ are calculated by taking the density function theory (DFT) method. It has been reported that the *d* orbitals of transition metal centers play a crucial role in the interaction with reaction intermediates.^[10]^ The change of *d* orbitals can modify the related electronic structures, which in turn affect the reaction energy barrier. For the polysulfide reduction catalysts, the effective electronic states of intermediates (Li_2_S_2_ and Li_2_S) are usually dominated by the *p* orbitals of sulfur.^[11]^ The hybridization between *d* orbitals of metal centers and *p* orbitals of sulfur will largely affect the catalytic activities. Therefore, the *d‐p* orbital hybridization between different FEs‐based SACs and Li_2_S_2_ are explored to predict their reaction free energies. The partial projected density of state (PDOS) of the FEs−N_4_ (Figure S1, Supporting Information) shows that the electronic states of *d* orbitals (Fe−N_4_) present an asymmetry that originated from the uneven distribution of electrons in spin up and spin down. In comparison, this asymmetry of *d* orbitals in Co−N_4_ is weaker and eventually disappears completely in the Ni−N_4_ center. The asymmetry of *d* orbitals for FEs−N_4_ will cause spontaneous spin polarization that can provide spin electrons, which is beneficial for bonding with polysulfide molecules.[^9b^, ^12^] In order to quantify the spin polarization of FEs‐based SAC, their spin density and magnetic moments are calculated. Figure 1a and Figure S2 (Supporting Information) shows that Fe−N_4_ possessed the largest spin density and magnetic moment of 1.91 μ_B_, suggesting its superior spin polarization degree compared to the Co−N_4_ and Ni−N_4_.

To confirm that the ability of chemisorbing polysulfides is related to the spin polarization degree, we further analyze the molecular orbitals of Li_2_S_2_, FEs−N_4_, and Li_2_S_2_−FEs−N_4_ (Figure S3, S4, Supporting Information). Results show that among FEs−N_4_, the Fe−N_4_ possessing most spin electrons, thus leading to less antibonding orbitals occupation in Li_2_S_2_−Fe−N_4_ and resulting in robust Fe−S interaction. Furthermore, the optimized adsorption configuration of Li_2_S_*x*_ (1≤*x*≤8) on FEs−N_4_ is considered (Figure S5, S6, Supporting Information). In the case of Fe−N_4_ and Co−N_4_, Li bond with the N/C atom and S bond with Fe or Co atom, respectively. While for the Ni−N_4_, there is no apparent interaction between S and Ni atoms. Different from Ni‐based compounds, it is the Li−N instead of S−Ni interaction promoting Ni−N_4_ absorbing Li_2_S_*x*_. The binding strength of Li_2_S_*x*_ with FEs−N_4_ sites (Figure 1b) shows that the Fe−N_4_ has the highest binding energies with S_8_, Li_2_S_6_, and Li_2_S_2_ of −0.96, −1.28, and −1.81 eV, respectively, in agreement with the results of the magnetic moment and spin polarization degree.

Considering that the conversion from Li_2_S_2_ to Li_2_S involves the dissociation of the S−S bond in Li_2_S_2_, the PDOS of Li_2_S_2_−FEs−N_4_ has been analyzed to reveal the relationship between the adsorption/reduction of Li_2_S_2_ on FEs−N_4_ surface and the electronic structure of the SACs. When the electronic states of FEs interact with the S atom, the hybridized energy levels will split into the anti‐bonding states (normally go across the Fermi level (*E*_f_)) and the bonding states (below the *E*_f_) (Figure S7, Supporting Information). The strength of the FEs−S interaction depends on the position of the antibonding state, and the higher the position of the anti‐bonding state, the stronger the interaction. As shown in Figure 1c, the *d*‐band centers (*ϵ*_d_) of FEs−N_4_ exhibit *ϵ*_d(Fe)_ (−0.79 eV)>*ϵ*_d(Co)_ (−1.74 eV)>*ϵ*_d(Ni)_ (−2.32 eV), meaning the *ϵ*_d(Fe)_ in Li_2_S_2_−Fe−N_4_ is closer to the Fermi level. This leads to a stronger interaction between Li_2_S_2_ and Fe−N_4_ than the Li_2_S_2_−Co−N_4_ and Li_2_S_2_−Ni−N_4_, which corresponds to the order of adsorption energy, thus consequently weakening the S−S bonds in Li_2_S_2_.^[13]^ Meantime, the Li_2_S_2_−Fe−N_4_ shows more electron occupation on its *d* orbitals near the Fermi level makes it more prone to accept or lose electrons, which suggests an excellent electron transfer ability of Fe sites and offers benefit to the following Li_2_S_2_ reduction reaction.^[14]^

Furthermore, an obvious charge redistribution between the Li_2_S_2_ and FEs−N_4_ can be observed in the diagrams of charge density differences. The number of charge transfers between the FEs and S bond has been calculated by Bader charge analysis (Figure 1d–f); there are 0.20, 0.18, and 0 |e| charges that transfer from S atom to FEs atoms, respectively, suggesting the strongest electron exchange between S and Fe atoms. To further learn the actual states of S−S interaction, the PDOS of S in Li_2_S_2_−FEs−N_4_ is analyzed. As shown in Figure 1g, the (*s p*) orbitals of S2 (the S that directly connects to Fe) match well with S1 in Li_2_S_2_−Ni−N_4_, indicating a strong S−S bond. This degree of matching is weakened in Li_2_S_2_−Co−N_4_ and Li_2_S_2_−Fe−N_4_, where the latter is particularly pronounced, implying a weakened S−S bond due to the transfer of internal charge from the S2 to Fe site.

We then calculate the bond lengths of S−S and FEs−S in Li_2_S_2_−FEs−N_4_ to reveal their internal interactions (Figure 1h). Notably, the S−S bond lengths show a trend of Fe−N_4_ (2.120 Å)>Co−N_4_ (2.103 Å)>Ni−N_4_ (2.101 Å), while the FEs−S bond lengths present an order of *d*_Fe−S_ (2.24 Å)<*d*_Co−S_ (2.31 Å)<*d*_Ni−S_ (3.29 Å), thus indicating a strong interaction and electron transfer within Fe−S, therefore effectively weakening the S−S bond in Li_2_S_2_. The free energy diagrams for the Li_2_S_2_−Li_2_S reduction catalysis in FEs‐based SAC show that the Fe−N_4_ site (0.56 eV) displays the lowest thermodynamic energy barrier compared to the Co−N_4_ (0.60 eV) and Ni−N_4_ (0.88 eV), respectively (Figure 1i). All the calculation results reveal that the Fe−N_4_ catalyst presents the easiest Li_2_S_2_−Li_2_S reduction conversion activity, indicating that the spin polarization is responsible for its enhanced FEs−S interaction and accelerated solid‐solid catalytic conversion, which will also be further confirmed by our following experimental results.

**Figure 2:**
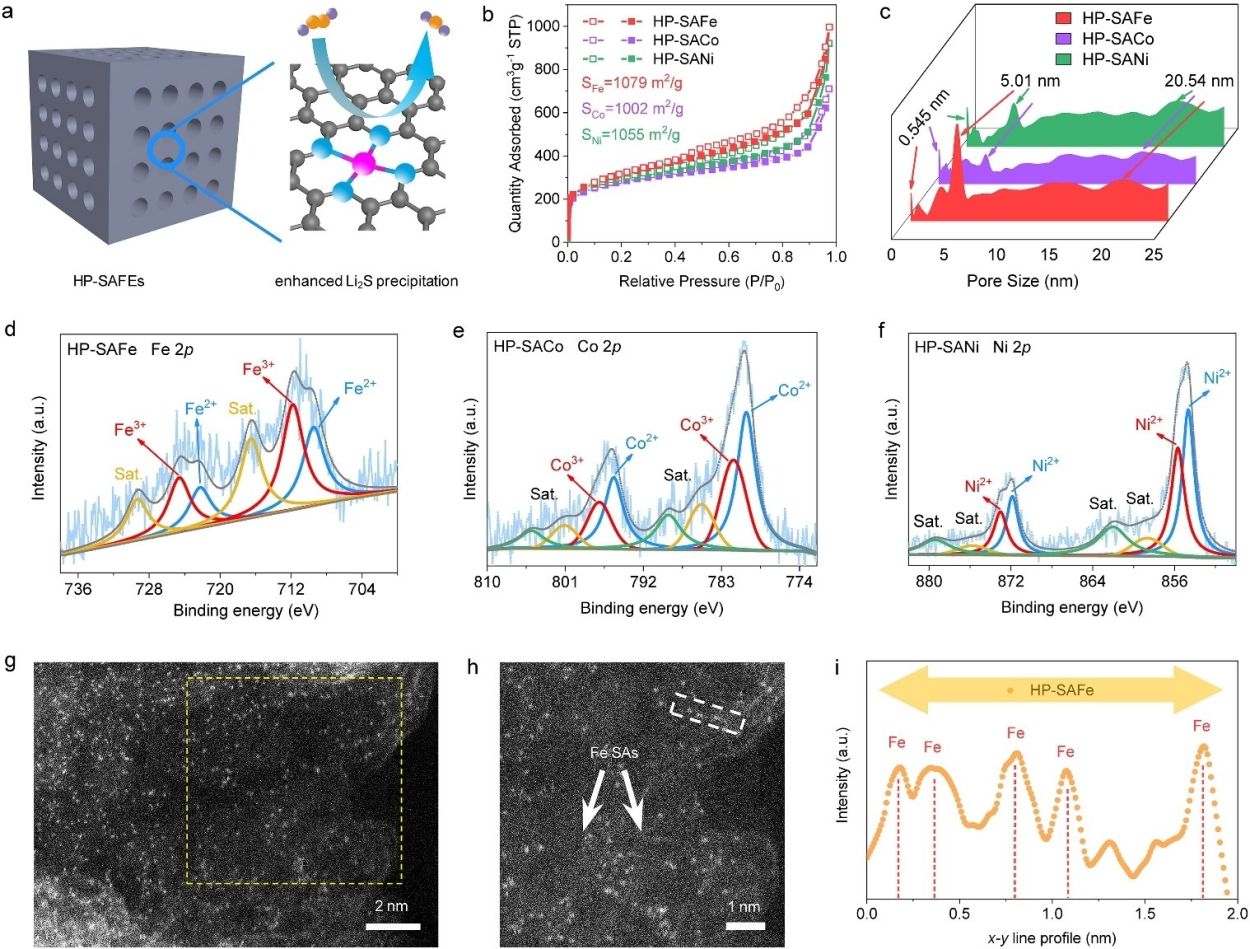
Structure analysis of HP‐SAFEs. a) The schematic porous structure and b) N_2_ adsorption‐desorption measurements and c) corresponding pore size distributions for HP‐SAFEs. d) XPS spectra of Fe 2p for HP‐SAFe, e) Co 2p for HP‐SACo, and f) Ni 2p for HP‐SANi. g) AC‐STEM image and h) the magnified image of HP‐SAFe. i) x‐y line scan profile, measured from (h). j) XANES spectra at the Fe K‐edge and k) their magnified image. l) Fourier transforms the EXAFS spectra for Fe K‐edge of Fe foil, Fe_2_O_3_, FePc, and HP‐SAFe. m) The corresponding EXAFS fitting curves of HP‐SAFe at R space. Wavelet transformation of Fe K‐edge EXAFS of n) Fe_2_O_3_, o) HP‐SAFe, and p) FePc, respectively.

To verify the above‐proposed mechanism, we have synthesized a series of HP‐SAFEs (Figure 2a) as Li_2_S_2_−Li_2_S reduction catalysts to assemble Li−S batteries by utilizing silica embedded nanocubic metal‐organic precursor with in situ FEs doping (Figure S8, Supporting Information). First, the precursors are pyrolyzed at 900 °C in the Ar atmosphere; after removing silica, secondary thermal treatment is conducted to obtain the HP‐SAFEs. All the synthesized HP‐SAFEs exhibit cubic morphologies with rough surfaces (Figure S9, Supporting Information); meantime, obvious mesoporous are found under scanning electron microscopy (SEM). The control sample of hierarchical porous N‐doped carbon without metal atoms doping (HP‐NC) is also prepared. The similar specific surface areas and pore structures of the HP‐SAFe (1079 m^2^ g^−1^), HP‐SACo (1002 m^2^ g^−1^), HP‐SANi (1056 m^2^ g^−1^), and HP‐NC (1130 m^2^ g^−1^) are validated by the N_2_ adsorption/desorption analysis (Figure 2b and c, Figure S10, Supporting Information).

To further explore the electronic structure and coordination environment of HP‐SAFEs, we first analyze the high‐resolution X‐ray photoelectron spectroscopy (XPS) of N 1s spectra. The result shows that pyridinic N in HP‐NC has a binding energy of 398.35 eV, while it shifts to 398.55 eV for HP‐SAFEs (Figure S11, Supporting Information), which suggests that the metal ions are bonded with pyridinic N to form the atomic metal‐N_*x*_ sites.^[15]^ Moreover, the XPS and inductively coupled plasma (ICP) confirms that HP‐SAFEs display similar metal contents of Fe (0.26 At. %, 1.09 wt %), Co (0.42 At. %, 1.15 wt %), and Ni (0.34 At. %, 0.85 wt %), respectively (Figure 2d–f, Table S2, Supporting Information). The Fe region scan shows two main peaks in the Fe *2p*_*3/2*_ at the binding energy of 709.20 and 711.70 eV, which correspond to the Fe^2+^ and Fe^3+^ oxidation states, respectively. Ni *2p* and Co *2p* spectra also present a similar oxidation state of Co^2+^/Co^3+^ (780.13 and 781.60 eV) and Ni^2+^ (854.66 and 855.67 eV).^[16]^ No zero‐valent metal peaks can be found for all the XPS spectra of HP‐SAFEs, thus indicating no metallic particles or clusters in these HP‐SAFEs.

**Figure 3:**
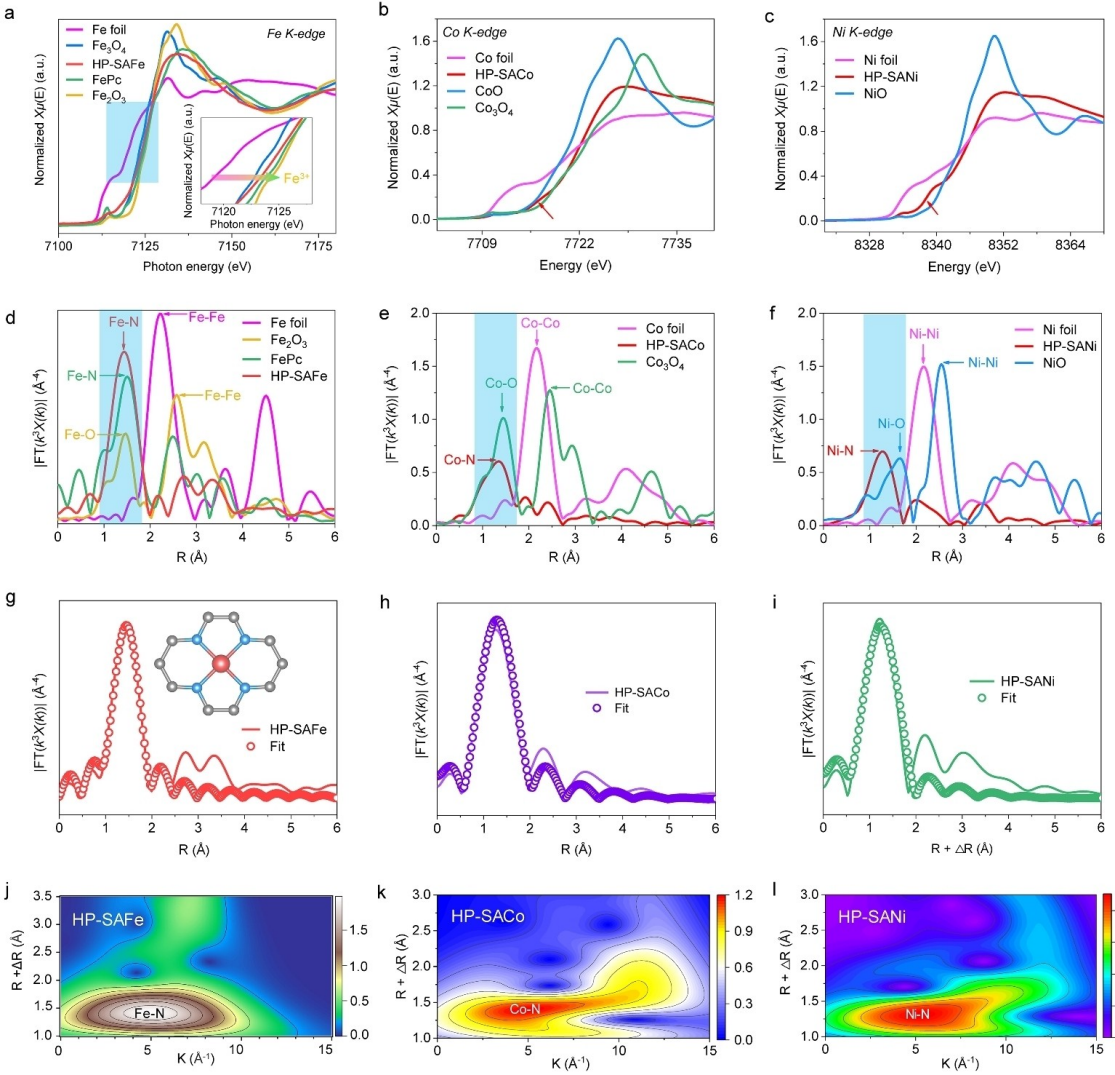
Configuration analysis of HP‐SAFEs. a) XANES spectra at the Fe K‐edge, b) Co K‐edge, c) Ni K‐edge. d) Fourier transforms the EXAFS spectra for Fe K‐edge of HP‐SAFe, e) Co K‐edge of HP‐SACo, f) Ni K‐edge of HP‐SANi. g) The corresponding EXAFS fitting curves of HP‐SAFe, h) HP‐SACo, i) HP‐SANi at R space. Wavelet transformation of j) Fe K‐edge EXAFS of HP‐SAFe, k) Co K‐edge EXAFS of HP‐SACo, and l) Ni K‐edge EXAFS of HP‐SANi, respectively.

The atomic‐scale structure of the representative HP‐SAFe is future observed under a spherical aberration‐corrected scanning transmission electron microscope (AC‐STEM). Figure 2g–i and Figure S14 (Supporting Information) show abundant bright dots, indicating the atomic distribution of Fe atoms in the porous carbon substrate; no Fe clusters or particles can be observed. The X‐ray absorption near‐edge structure (XANES) and extended X‐ray absorption fine structure (EXAFS) spectroscopy are performed to further reveal the coordination environment and valence state of Fe atoms. The XANES curves at the Fe K‐edge show that the position of HP‐SAFe is located between those of Fe_3_O_4_ and Fe_2_O_3_, corroborating the valence state is between Fe^2+^ and Fe^3+^, meantime HP‐SACo corroborates the valence state between Co^2+^ and Co^3+^, and HP‐SANi between Ni^0^ and Ni^2+^(Figure 3a–c). Fourier‐transforms (FTs) and wavelet‐transforms (WTs) images from the EXAFS spectra of HP‐SAFEs depict that the FEs is found to be bonded as FEs−N/C/O.^[17]^ No obvious metal peak in the FTs spectrum of HP‐SAFEs is observed, revealing their atomic dispersion (Figure 3d–l). The least‐squares EXAFS fitting parameters at the Fe K‐edge of HP‐SAFe show the Fe−N bond length of 1.99 Å and coordination number of 4.1, which are very similar to that determined for FePc (2.01 Å, *n*=4.0) (Figure 3g, Figure S15 and Table S3, Supporting Information). Meanwhile, HP‐SACo and HP‐SANi show the Co−N and Ni−N bond length of 1.75 Å and 1.71 Å with coordination number of 4.0 and 4.1, respectively (Figure 3h and i). Based on the above analysis, we suggest that the isolated FEs atoms in HP‐SAFEs are tetra‐coordination by N atoms and form a typical FEs−N_4_ structure in the HP‐NC matrix.

**Figure 4:**
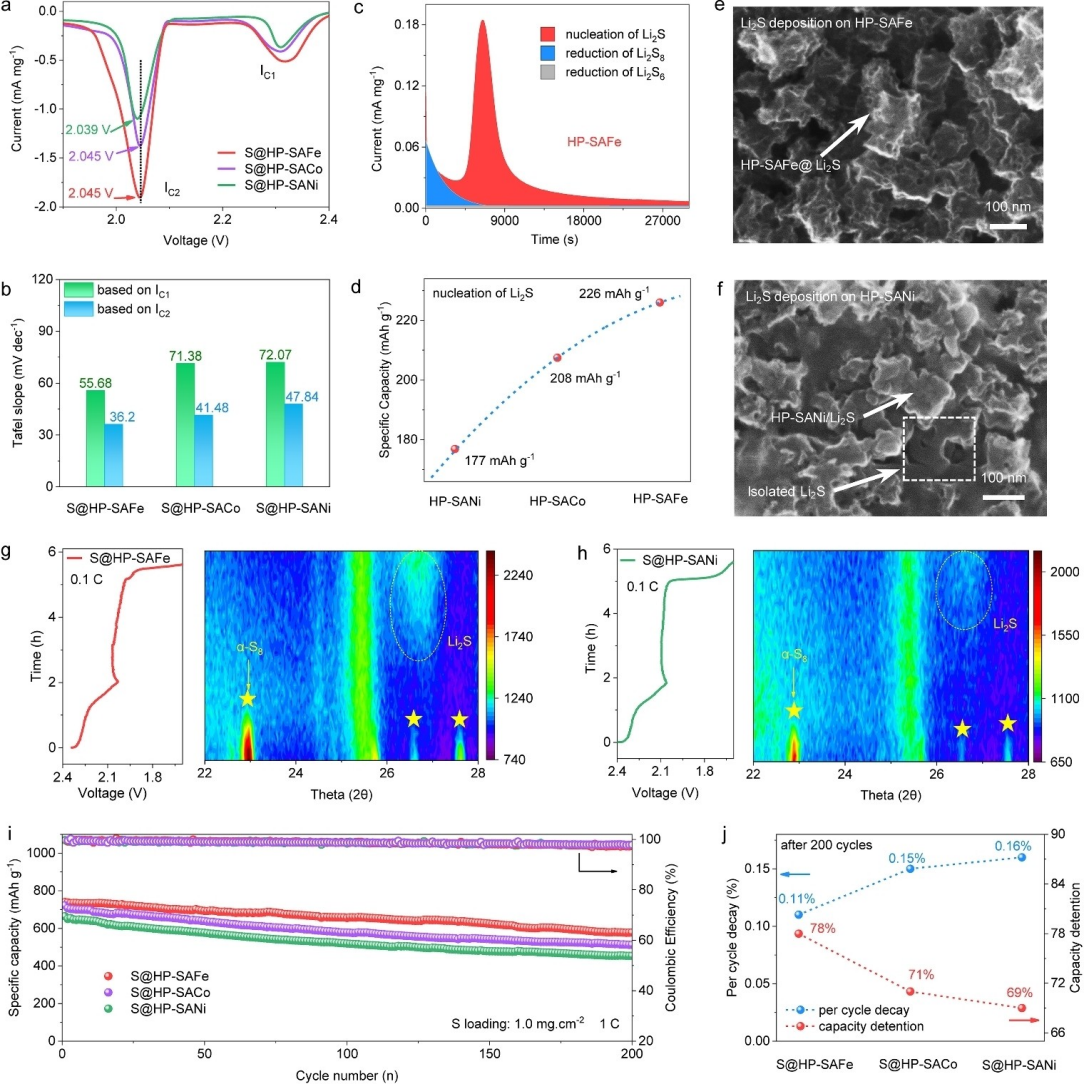
Cathode performances of synthesized HP‐SAFEs. a) CV tests of S@HP‐SAFEs at 0.1 mV s^−1^. b) Corresponding Tafel plots for S@HP‐SAFEs. c) Potentiostatic discharge curves for HP‐SAFe. d) Specific capacities for the step of Li_2_S deposition on the HP‐SAFEs. e) SEM images of electrodeposition tests on the HP‐SAFe and f) HP‐SANi. Galvanostatic discharge curve and corresponding in situ XRD patterns for g) S@HP‐SAFe and h) S@HP‐SANi electrode. i) Cycling tests of S@HP‐SAFEs at 1 C with the sulfur loading of 1.0 mg cm^−2^. j) The capacity decay per cycle and capacity detention of S@HP‐SAFEs after 200 cycles at 1 C.

After validation that the HP‐SAFEs display similar morphologies, surface areas, pore structures, and metal contents, it is reliable to use these synthesized HP‐SAFEs to explore and compare the Li_2_S_2_−Li_2_S reduction activities of SAFEs experimentally. After heating the mixture of HP‐SAFEs and sulfur at 155 °C, the resulted S@HP‐SAFEs containing 80 wt % sulfur (Figure S16, Supporting Information) are used as cathodes in Li−S batteries. The cubic morphology of S@HP‐SAFEs can be well‐maintained after sulfur is immitted (Figure S17, S18, Supporting Information). The electrocatalysis of polysulfides and conversion of insoluble Li_2_S_2_ to Li_2_S is explored by cyclic voltammetry (CV); all the curves of S@HP‐SAFe, S@HP‐SACo, and S@HP‐SANi show clearly two reduction peaks (I_C1_ and I_C2_) that are ascribed to the reduction of S_8_ (I_C1_) and whereupon conversion to Li_2_S (I_C2_), respectively (Figure 4a). The peak current densities of I_C2_ follow the order of S@HP‐SAFe (−1.91 mA mg^−1^)>S@HP‐SACo (−1.37 mA mg^−1^)>S@HP‐SANi (−1.10 mA mg^−1^), which suggests that the S@HP‐SAFe cathode can effectively facilitate the Li_2_S deposition.^[18]^ The determination of Tafel slopes offer the kinetic parameters to illustrate the catalytic activities of the polysulfide electrocatalyst at different voltage intervals. Figure 4b and Figure S20 (Supporting Information) show that the S@HP‐SAFe delivers the smallest Tafel slopes (55.68 and 36.20 mV dec^−1^) at both two reduction stages, indicating its faster reaction kinetics than S@HP‐SACo and S@HP‐SANi.

More importantly, the reduction kinetics of the liquid‐solid reaction from polysulfides to Li_2_S are investigated to reveal the nucleation behaviors of solid‐state Li_2_S on the electrode surface, which is proceeded with a potentiostatic method at 2.05 V after the first galvanostatic discharging process at a current of 0.112 mA. The integral areas of the current peaks that corresponds to the nucleation of Li_2_S are calculated based on Faraday's law.^[19]^ The value for the HP‐SAFe is 226 mAh g^−1^, which is much higher than that for HP‐SACo (208 mAh g^−1^) and HP‐SANi (177 mAh g^−1^), thus suggesting that HP‐SAFe has the fastest catalytic deposition ability of Li_2_S as depicted by our theoretical calculation (Figure 4c and d, Figure S21, Supporting Information). The electrodeposition morphology of the Li_2_S on cathodes is investigated by SEM and energy‐dispersive spectroscopy (EDS) mapping analysis. It is found that Li_2_S is uniformly deposited on the surface of HP‐SAFe with approximately 100 % coverage, which is attributed to the low energy barriers of Li_2_S nucleation and growth on HP‐SAFe (Figure 4e, Figure S22, Supporting Information). While the HP‐SANi exhibits an insufficient coverage on the cathode surface with isolated Li_2_S deposition (Figure 4f, Figure S22, Supporting Information).

The boosted catalytic deposition of Li_2_S on HP‐SAFe is directly monitored by the in situ X‐ray diffraction (XRD) technique, which provides clear insights into the deposition activity of Li_2_S. The cell is first discharged at a rate of 0.1 C, during which a series of XRD spectra are recorded at different voltages. The contour pattern of the S@HP‐SAFe cathode shows an obvious transition signal of crystalline α‐S_8_ to Li_2_S during the discharge process (Figure 4g). While the S@HP‐SANi cathode displays a much weaker Li_2_S signal at the end of the discharge process (Figure 4h), suggesting a poor polysulfides conversion property. After disclosing the order of Li_2_S_2_−Li_2_S reduction ability for S@HP‐SAFEs, we also perform the long‐term cycling experiments at a high rate of 1 C to compare the cathode performances (Figure 4i and j). The S@HP‐SAFe exhibits the best cycling stability with 97.6 % Coulombic efficiency and reversible specific capacities of 578 mAh g^−1^ after 200 cycles (0.11 % decay per cycle), which is higher than those of the S@HP‐SACo (512 mAh g^−1^, 0.15 % decay per cycle) and S@HP‐SANi (454 mAh g^−1^, 0.16 % decay per cycle).

**Figure 5:**
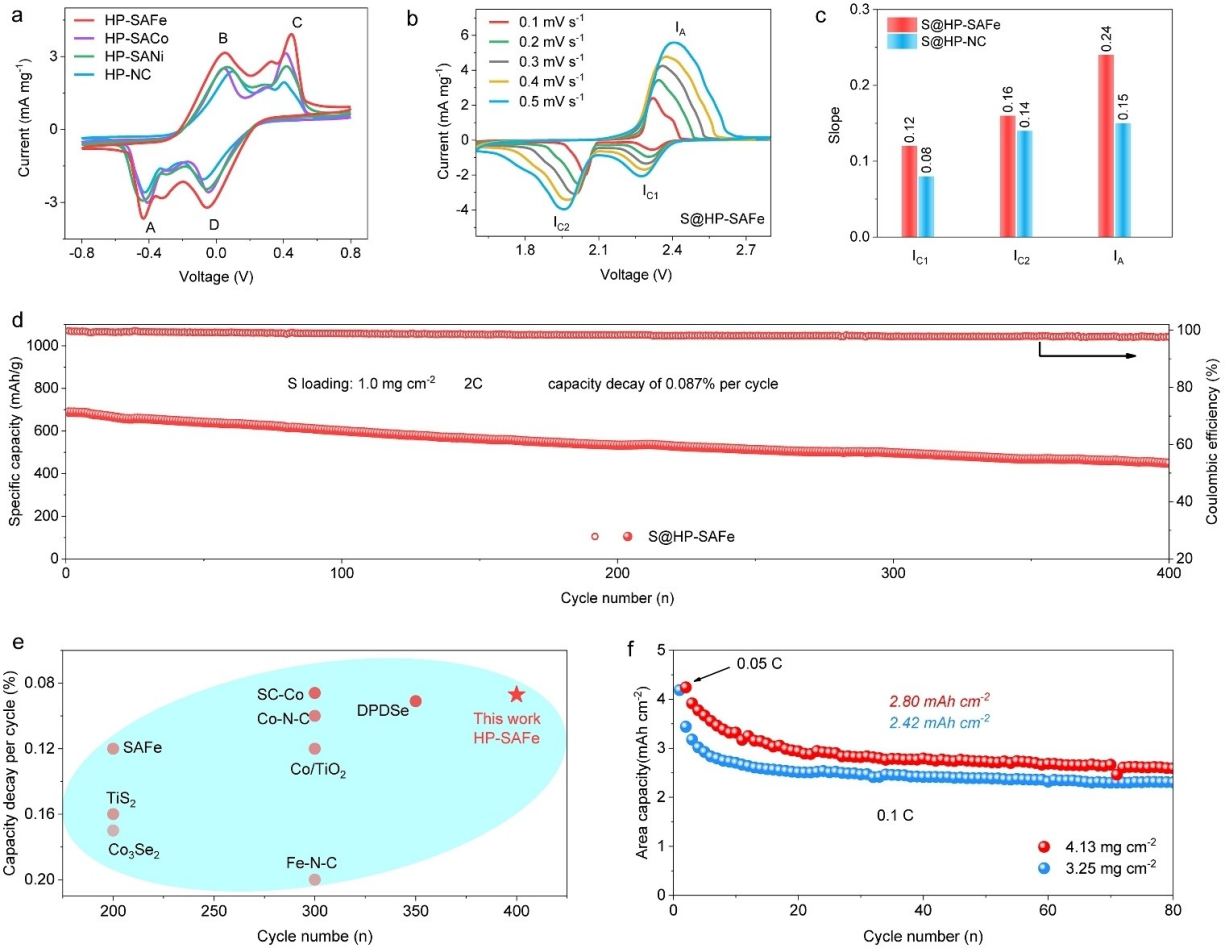
Electrochemical performances of S@HP‐SAFe. a) CV profiles of symmetric cells employing HP‐SAFe and HP‐NC with Li_2_S_6_ at a scanning rate of 3 mV s^−1^. b) CV curves of S@HP‐SAFe electrodes at 0.1–0.5 mV s^−1^. c) The slope statistics from (b). d) Cycling stability of HP‐SAFe‐based sulfur cathodes at 2 C with a sulfur loading of 1.0 mg cm^−2^. e) Performance comparisons with recent works on polysulfide catalysts‐based cathodes in Li−S batteries for long‐term cycling. f) Cycling stability of HP‐SAFe‐based sulfur cathodes at 0.1 C with the sulfur loading of 3.25 and 4.13 mg cm^−2^.

Since the HP‐SAFe has displayed the best performances on the Li_2_S_2_−Li_2_S reduction catalysis by both theoretical and experimental analysis. To explain the importance and kinetics of atomic Fe sites in Li−S batteries, we then take a further analysis on the catalytic role and mechanisms of HP‐SAFe by using the HP‐NC as the control sample. The kinetics of polysulfide reductions in the liquid phase is conducted by CV of symmetric cells with identical working and counter electrodes in 0.5 M Li_2_S_6_ electrolyte (Figure 5a). The CV of HP‐SAFe exhibits four pronounced reduction/oxidation peaks located at −0.44 V (peak A), 0.05 V (peak B), 0.45 V (peak C), and −0.05 V (peak D). These peaks can be assigned to the electrochemical reactions of Li_2_S_6_ on the electrodes, including the reduction of Li_2_S_6_ to Li_2_S (peak A), the reconstitution of Li_2_S_6_ by the oxidation of Li_2_S (peak B), the oxidation of Li_2_S_6_ to generate S_8_ (peak C), and the reduction of S_8_ to Li_2_S_6_ (peak D).^[20]^ The CV of HP‐NC, HP‐SACo, and HP‐SANi also show four reduction/oxidation peaks, but their peak currents are significantly lower than the HP‐SAFe. Thus, the CV results clearly indicate that the HP‐SAFe electrode provides the best electrochemical kinetics for polysulfide conversion.^[21]^

In order to investigate the lithium diffusion properties, the CV curves of S@HP‐SAFe and S@HP‐NC cathodes containing similar amounts of sulfur under different scanning rates are investigated (Figure 5b). All reduction and oxidation peak currents are linear with the square root of scanning rates (Figure S24, Supporting Information), from which the lithium diffusivity could be estimated according to the classical Randles Sevcik equation.[^20^, ^22^] As shown in Figure 5c, the slopes of the S@HP‐SAFe electrode are higher than that of the S@HP‐NC electrode in all sulfur conversion steps, implying the faster lithium diffusivity and better polysulfide reaction kinetics.[^19b^, ^23^]

To confirm the application potential of the S@HP‐SAFe cathode, we then conduct the long‐term cycling experiments at the charge/discharge rate of 0.5 C (Figure S26, Supporting Information) and 2 C. At 0.5 C, apparently, the S@HP‐SAFe exhibits a much higher initial capacity of 882.4 mAh g^−1^ and per cycle capacity decay of 0.18 % (150 cycles) than those of S@HP‐NC (774.1 mAh g^−1^ and 0.25 %, respectively). Moreover, when subjected to a high‐rate cycling evaluation at 2 C, the S@HP‐SAFe shows an initial capacity of about 700 mAh g^−1^ and a small capacity decay of 0.087 % per cycle over 400 cycles (Figure 5d), which is comparable with the state‐of‐the‐art polysulfide catalysts (Figure 5e, and Table S4, Supporting Information). As shown in Figure S27, S28 (Supporting Information), the S@HP‐SAFe exhibits a series of advantageous discharge capacities of 1542, 899, 761, 689, and 639 mAh g^−1^ when cycled at 0.1, 0.2, 0.5, 1.0, and 2.0 C, respectively. When the specific current is switched back to 0.1 C, a high discharge capacity of 794 mAh g^−1^ can be maintained. To further demonstrate the excellent potential of the as‐designed S@HP‐SAFe cathode for constructing practically viable Li−S batteries, we prepare two composite cathodes with very high sulfur loading of 3.25 and 4.13 mg cm^−2^. Notably, the corresponding S@HP‐SAFe cathodes exhibit high reversible average areal capacity of 2.42 and 2.80 mAh cm^−2^ under steady cycling for 80 cycles at 0.1 C, thus achieving a stable polysulfide reduction electrochemistry (Figure 5f).

**Figure 6:**
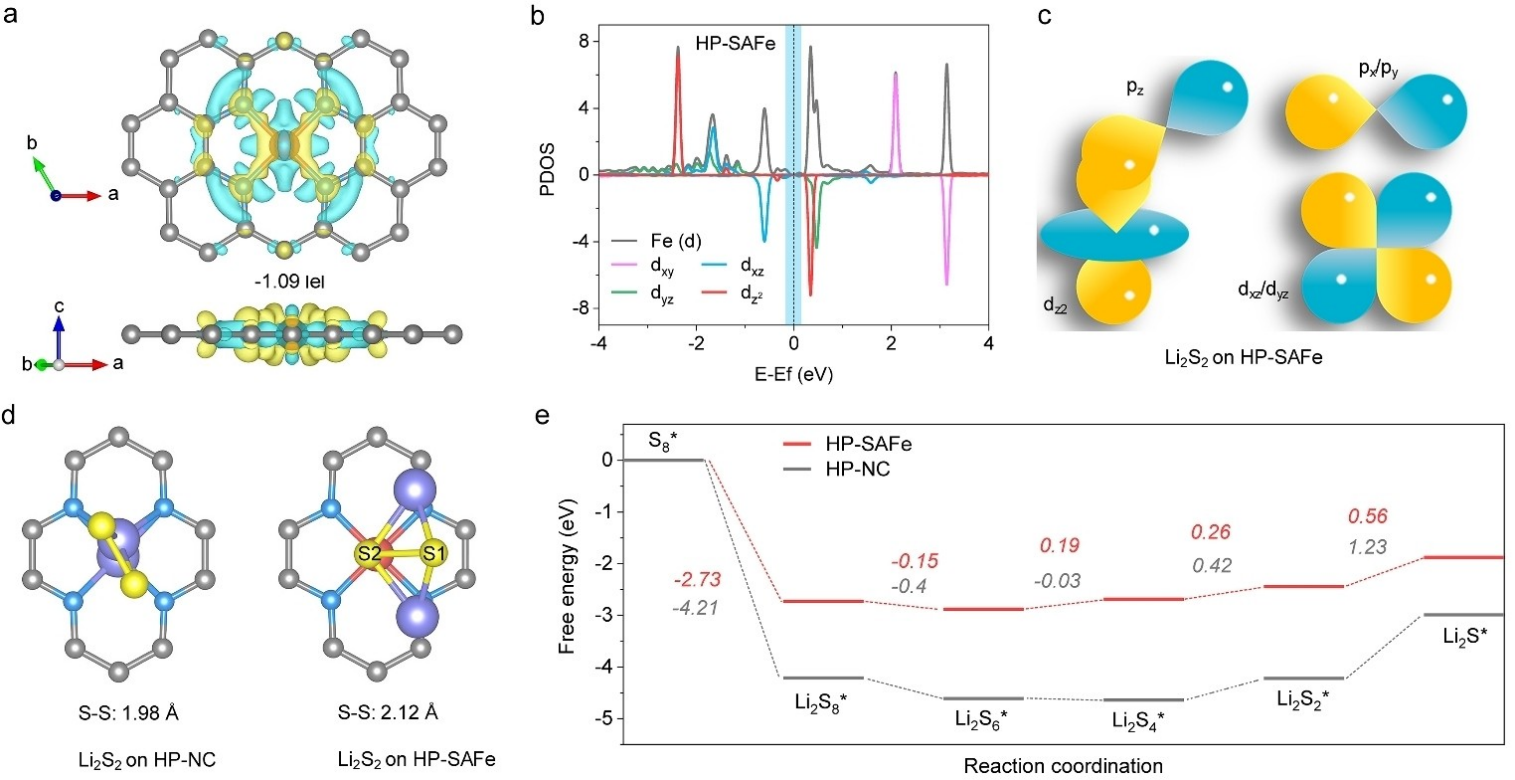
DFT calculations on the polysulfide reduction catalysis of HP‐SAFe. a) Differential charge density of HP‐SAFe, yellow represents electron accumulation, and cyan represents electron reduction. b) The calculated PDOS of HP‐SAFe. c) The orbital interaction of Li_2_S_2_ on HP‐SAFe. d) The optimized structures of Li_2_S_2_ on HP‐NC and HP‐SAFe, respectively. e) Free energy profiles for the catalytic reduction of polysulfide intermediates on HP‐NC and HP‐SAFe.

To understand the reason for the improved reduction reaction kinetics of the HP‐SAFe toward the catalytic conversion of polysulfide intermediates, we have conducted a comprehensive first‐principles calculation. As shown in Figure 6a, the Fe center presents a Bader charge of −1.09|e|, which changes the originally electroneutral Fe into positively charged. Meanwhile, the charge density differences show that there is charge transfer from Fe to N atoms, thus presenting a state of charge depletion on top of the metal center, which may offer favorable interaction to the polysulfide intermediates. To further understand the electronic structures, the PDOS of HP‐SAFe has been calculated and shown in Figure 6b. The $d_{z^{2}}$ orbital perpendicular to the basal plane remains unoccupied near the Fermi level.^[24]^ Meantime, the HP‐SAFe demonstrates a nearly negligible band gap between the conduction band and the valence band; thus, it may boost the electron transfer during reduction catalysis.^[14]^

Then, the PDOS of Li_2_S_2_ on HP‐SAFe has also been investigated; the *p_z_* and *p_x_*/*p_y_* orbitals of sulfur can hybridize with the $d_{z^{2}}$ and *d_xz_*/*d_yz_* orbitals of iron, respectively (Figure 6c, Figure S29, Supporting Information). This suggests the strong *d‐p* coupling and electronic interaction in the Fe−S pair. The optimized adsorption structures of Li_2_S_2_ on HP‐NC and HP‐SAFe are then shown in Figure 6d, where the S−S bond lengths in Li_2_S_2_ are 1.98 Å and 2.12 Å, respectively, suggesting the S−S bond in HP‐SAFe is easier to break. Subsequently, the relative free energy landscape for the discharging process from S_8_ to Li_2_S on the HP‐NC and HP‐SAFe surface have clearly revealed that the Li_2_S_2_−Li_2_S reduction catalysis exhibits a larger thermodynamic energy barrier compared to the other steps on both substrates, suggesting that the Li_2_S deposition process was the rate‐determine step during discharging (Figure 6e).^[25]^ The HP‐SAFe surface shows an energy barrier of 0.56 eV in the Li_2_S_2_−Li_2_S reduction process, which is significantly lowered than the HP‐NC (1.23 eV). With these results, we can conclude that the Li_2_S_2_ reduction and Li_2_S deposition process is thermodynamically favorable on the HP‐SAFe surface, thus resulting in a much higher cathode performance than the others.^[7]^

## Conclusion

We have demonstrated for the first time that the fundamental origin of insoluble Li_2_S_2_−Li_2_S reduction catalysis in FEs‐based single‐atom materials (Fe, Co, Ni) should be related to their spontaneous spin polarization (Fe−N_4_>Co−N_4_>Ni−N_4_). Among FEs−N_4_, the Fe−N_4_ possessing most spin electrons undoubtedly result in robust Fe−S interaction in Li_2_S_2_−Fe−N_4_, and then weaken the S−S bond, accelerating insoluble Li_2_S_2_−Li_2_S reduction catalysis. As a result, cathode with HP‐SAFe exhibits the fastest deposition kinetics of Li_2_S (226 mAh g^−1^) and the lowest thermodynamic energy barriers (0.56 eV). Remarkably, the corresponding S@HP‐SAFe cathodes exhibit high reversible average areal capacity of 2.8 mAh cm^−2^ at sulfur loading of 4.1 mg cm^−2^ for 80 cycles, suggesting its practical opportunities towards high‐energy and long‐life Li−S batteries.

## Conflict of interest

The authors declare no conflict of interest.

1

## Supporting information

As a service to our authors and readers, this journal provides supporting information supplied by the authors. Such materials are peer reviewed and may be re‐organized for online delivery, but are not copy‐edited or typeset. Technical support issues arising from supporting information (other than missing files) should be addressed to the authors.

Supporting Information

## Data Availability

The data that support the findings of this study are available from the corresponding author upon reasonable request.
