# Supplementary material for: Origin and Acceleration of Insoluble Li2S2−Li2S Reduction Catalysis in Ferromagnetic Atoms‐based Lithium‐Sulfur Battery Cathodes
Source: Angew Chem Int Ed Engl. 2022 Dec 1;62(1):e202215414. doi: 10.1002/anie.202215414 (PMC10107143; doi:10.1002/anie.202215414)
Supplement: Supplementary file 1 — Supporting Information [file ANIE-62-0-s001.pdf]

Angewandte Chemie
Eine Zeitschrift der Gesellschaft Deutscher Chemiker

Supporting Information

Origin and Acceleration of Insoluble Li_2S_2–Li_2S Reduction Catalysis in Ferromagnetic Atoms-based Lithium-Sulfur Battery Cathodes

R. Yan, Z. Zhao, M. Cheng, Z. Yang, C. Cheng, X. Liu*, B. Yin, S. Li**

Supporting Information

Experiment Section

1. Materials and reagents

2-Dimethylimidazole (98%), Zinc nitrate hexahydrate (99%), Iron nitrate nonahydrate (98%), Hexadecyltrimethylammonium Bromide (CTAB, 99%), Ammonium bifluoride (NH_4HF_2, 98%), Sublimed sulfur (⩾ 99%), Lithium sulfide (99.98%), N-Methyl-2-pyrrolidone (NMP, 99.9%) are obtained from Aladdin (Shanghai, China). LUDOX®AS-40 colloidal silica (40 wt%) is purchased from Sigma. Ketjen Black, Polyvinylidene fluoride (PVDF, 99.5%), tetraethylene glycol dimethyl ether (TEGDME, 99%), lithium-sulfur battery electrolyte (1.0 M LiTFSI in DOL:DME=1:1 Vol% with 1.0% $LiNO_3$) are provided by KeLuDe. All chemical reagents are used as received without further purification. Deionized (DI) water and pure water are produced in our lab.

2. Synthesis of HP-NC and HP-SAFEs

2-Dimethylimidazole (5.65 g) and LUDOX®AS-40 colloidal silica (1 mL) are dissolved in deionized water (80 mL) under magnetic stirring. In addition, $Zn(NO_3)_2{\cdot}6H_2O$ (0.362 g) and

cetyltrimethyl ammonium bromide (CTAB) (10 mg) are dissolved in deionized water (20 mL), then added into 2-Dimethylimidazole solution to react and keep stirring for 1 h at room temperature. The products are collected by centrifugation and washed several times with deionized water and ethanol to remove the residual reactants to gain SiO_2@ZIF-8. Furthermore, the SiO_2@FEs/ZIF-8 is prepared similarly to SiO2@ZIF-8, except that additional Fe^{3+}/Co^{2+}/Ni^{2+} is added to the solution of $Zn(NO_3)_2·6H_2O$ with molar ratio n_{Zn}:n_{FEs} = 40:1. Next, the obtained SiO_2@ZIF-8 and SiO_2@FEs/ZIF-8 are pyrolyzed in Ar at 900 °C for 2 h followed by a etch with 4 M NH_4HF_2. Finally, the etched productions are pyrolyzed again in Ar at 900 °C for 2 h to obtain HP-NC and HP-SAFEs.

3. Materials Characterization

The scanning electron microscope (SEM) morphology of the synthesized materials is examined using a JSM-7500F SEM microscope (JEOL, Japan), and transmission electron microscopy (TEM) images are obtained via a Tecnai FEI Talos F200X TEM microscope (FEI Ltd., USA) operated at 200 kV). X-ray diffraction (XRD) pattern present the crystal phase state via a DX-2700BH multipurpose X-ray diffractometer (Haoyuan Instrument) with Cu radiation at a voltage of 40 kV. *In situ* XRD is performed using an *in situ* cell purchased from the Beijing Scistar Technology Co. Ltd., and the spectra are acquired during galvanostatic charge-discharge testing at 0.1 C. The N_2 adsorption/desorption isotherms are obtained by the Ankersmid Belsorp-Max and based on the Brunauer-Emmett-Teller (BET) to assess the surface area and pore size distribution, respectively. The surface elemental composition and all binding energies are measured by X-ray photoelectron spectroscopy (XPS, ESCAL 250) with Al Kα monochromatic X-ray sources.

4. Electrochemical characterization

Standard CR2032-type coin cells are assembled in an Ar-filled glove box with oxygen and moisture content below 1 ppm. The sulfur composite (S@HP-SAFEs), Ketjen Black, and polyvinylidene fluoride (PVDF) are well-mixed with a mass ratio of 8:1:1 in N-Methyl-2-pyrrolidone (NMP) to form the slurry. The composite electrodes are fabricated by coating the slurry on aluminum foil. The lithium foil is used as a counter and reference electrode, while the Celgard 2500 is used as the separator. The component of electrolyte is 1.0 M LiTFSI in DOL:DME=1:1 Vol% with 1.0%

$LiNO_3$ and the electrolyte/sulfur (E/S) is to be 20 μL mg^{-1}. Land 2001A battery testing system is used for the cycling and rate performance between 1.6 and 2.8 V (vs. Li^+/Li). During the cycle test, the maximum capacity after activation is used as the initial capacity. Cyclic voltammetry (CV) measurement is tested by a CHI600E electrochemical workstation.

5. Li_2S nucleation experiments

S and Li_2S (molar ratio of 7:1) and 1.0 M LiTFSI in tetraethylene glycol dimethyl ether solution are dissolved by stirring for 24 h to obtain the Li_2S_8 solution (0.25 mol L^{-1}). HP-SAFEs/HP-NC is used as working electrodes. 20 μL of Li_2S_8 solution is deposited onto the working electrode, and then 20 μL of a 1.0 M LiTFSI solution without Li_2S_8 is dropped onto the lithium anode. The batteries are discharged to 2.06 V at 0.112 mA and then kept potentiostatically at 2.05 V for Li_2S to nucleate and grow until the current fell below 10^{-5} A. The nucleation rate of Li_2S on the substrates is calculated based on Faraday's law.

6. Symmetrical cell assembly and measurements

Symmetric electrochemical cells are assembled by two identical electrodes. The electrode is prepared by mixing HP-SAFEs/HP-NC, Ketjen Black, and PVDF with the mass ratio of 8:1:1 in NMP, followed by coating the slurry on the Al foil. The Celgard 2500 is used as the separator. 0.5 M Li_2S_6 electrolyte is prepared by dissolving an appropriate amount of sulfur and Li_2S in the blank electrolyte (1 M LiTFSI in DOL/DME (1:1 by volume)) and stir at 70 ºC in an Ar-filled glove box overnight. 40 μL 0.5 M Li_2S_6 electrolyte is dropped in the CR2032 coin cell. CV is tested at 3 mV s^{-1} between -0.8 to 0.8 V on a CHI600E electrochemical workstation.

7. DFT calculations

All theoretical calculations are performed using DFT, as implemented in the Vienna ab initio simulation package (VASP).[1] The core electrons are described using the spin-polarized projector augmented wave (PAW) method,[2] and the electron exchange and correlation energy is treated within the generalized gradient approximation in the Perdew-Burke-Ernzerhof functional (GGA-PBE).[3] The valence states of all atoms are expanded in a plane-wave basis set with a cutoff energy of 500 eV. The

convergence criteria for the electronic self-consistent iteration and force are set to 10^{-5} eV, and 0.02 eV/Å with a Gamma centered 2 × 2 × 1 K-points. Denser 8 × 8 × 1 K-points are used for the density of states (DOS) computations. In addition, the Van der Waals interactions are included during all calculations using DFT-D3.[4] Slab model is constructed with a vacuum layer of 20 Å in the z direction to avoid the interaction between neighboring images. The charge density differences are evaluated using the formula $\Delta\rho = \rho_{A+B} - \rho_A - \rho_B$, where ρ_X is the electron density of X. Atomic charges are computed using the atom-in-molecule (AIM) scheme proposed by Bader.[5]

To quantitatively describe the binding ability of loading materials, the binding strength is defined as:

$$E_{ads} = E_{adsorbate/substrate} - E_{adsorbate} - E_{substrate}$$

where $E_{adsorbate/substrate}$, $E_{substrate}$, and $E_{adsorbate}$ represent the total energy of substrate with adsorbed species, the clean substrate, and the molecule in the gas phase, respectively. To explore the catalytic effect, the free energy evolution for the S_8, Li_2S_8, Li_2S_6, Li_2S_4, Li_2S_2, and Li_2S conversion are calculated.[6] The initial free energy for the adsorbed S_8 is set to zero while the Gibbs free energy for the intermediate step Li_2S_n (n = 2, 4, 6, 8) can be defined as:

$$G_{Li_2S_n} = E_{Li_2S_n} - n\cdot E_{S_8}/8 - 2\mu_{Li}$$

where $E_{Li_2S_n}$ and E_{S_8} are the energy of adsorbed Li_2S_n and S_8, which can be expressed as $E_{Li_2S_n/substrate}$-$E_{substrate}$ and $E_{S_8/substrate}$-$E_{substrate}$, respectively. μ_{Li} represents the chemical potential of the lithium bulk.

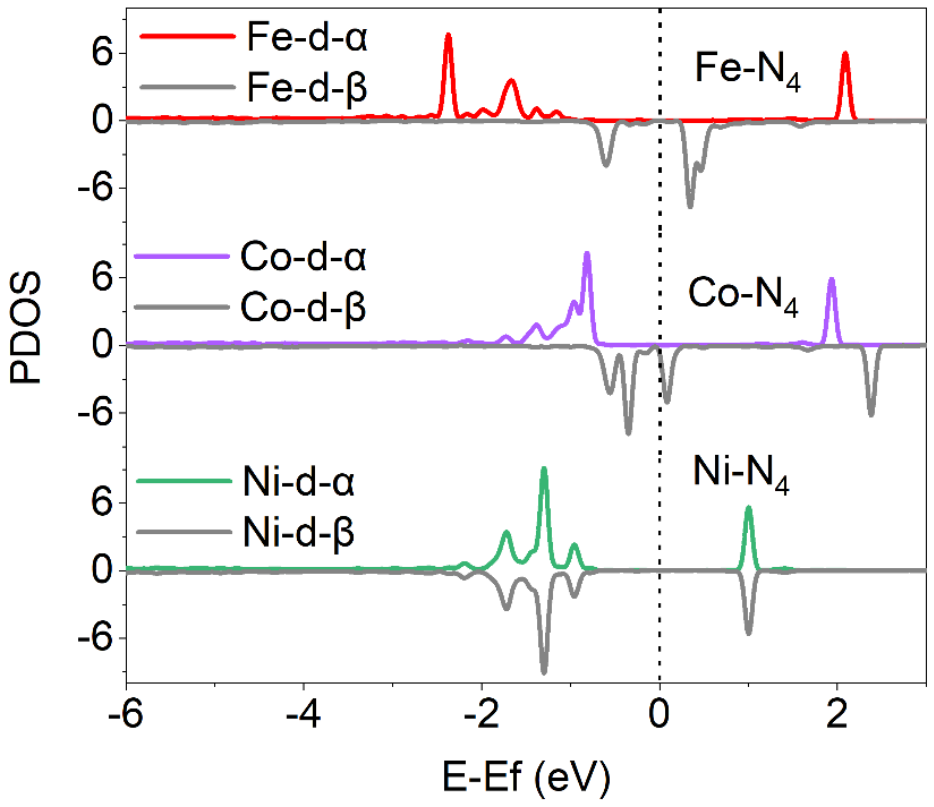

Figure S1. The PDOS of $Fe\text{-}N_4$, $Co\text{-}N_4$, and $Ni\text{-}N_4$.

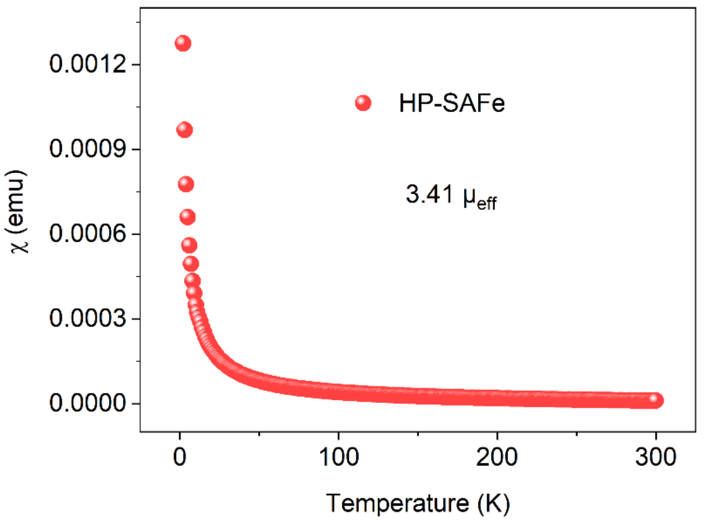

Figure S2. Magnetic susceptibility of HP-SAFe.

For insight into the electronic structure of representative HP-SAFe, the zero-field cooling (ZFC) temperature-dependent magnetic susceptibility curve is investigated. The calculated effective magnetic moment of HP-SAFe is 3.41 μ_{eff}, and the number of unpaired d electron (n) of HP-SAFe is about 2.5, which is consistent with our calculated result (2 unpaired electrons).

The effective magnetic moment (μ_{eff}) correlates with the number of unpaired electron (n) via the following equation:

$$\chi_m = M \times M_r/(H \times m)$$

$$2.828(\chi_m T)^{1/2} = \mu_{eff} = (n(n+2))^{1/2}$$

M: magnetic moment of the sample, the unit is emu; M_r: the molar mass of the sample; H: the applied magnetic field.

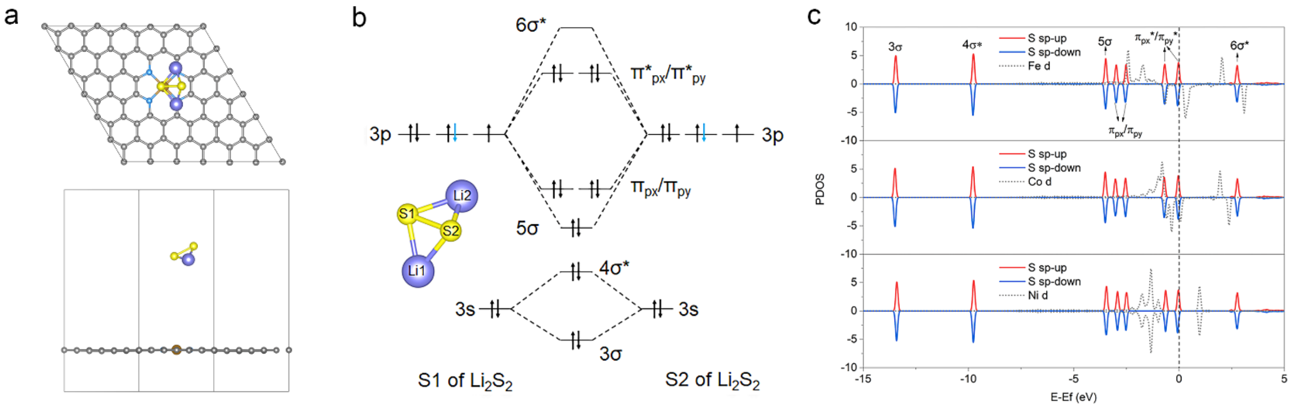

Figure S3. Electronic structure analysis of Li_2S_2 and FEs-N_4 without interaction. a) The optimized model of Li_2S_2 and FEs-N_4 without interaction. b) The molecular orbital diagram of typical Li_2S_2 molecule, in which the blue arrow represents one electron transferred from Li to S atom. c) The PDOS of the model a), where the molecular orbital arrangement of S corresponds to b).

To disclose the correlation between the spin polarization of FEs-N_4 and their activity for insoluble Li_2S_2-Li_2S reduction catalysis, the molecular orbital interaction between Li_2S_2 and FEs-N_4 must be first analyzed. As shown in Figure S3a, we take the structure of Li_2S_2 and FEs-N_4 without interaction as the research object. Based on the identification of the molecular orbital arrangement of individual Li_2S_2 molecules (Figure S3b), we reasonably acquire definite pDOS of S and FEs in individual Li_2S_2 and FEs-N_4 (Figure S3c). Clearly, the π/π^* orbitals of S tend to overlap with the d orbital of FEs, suggesting that Li_2S_2 interacts with FEs-N_4 primarily through the π/π^* orbitals of S and the d orbital of FEs.

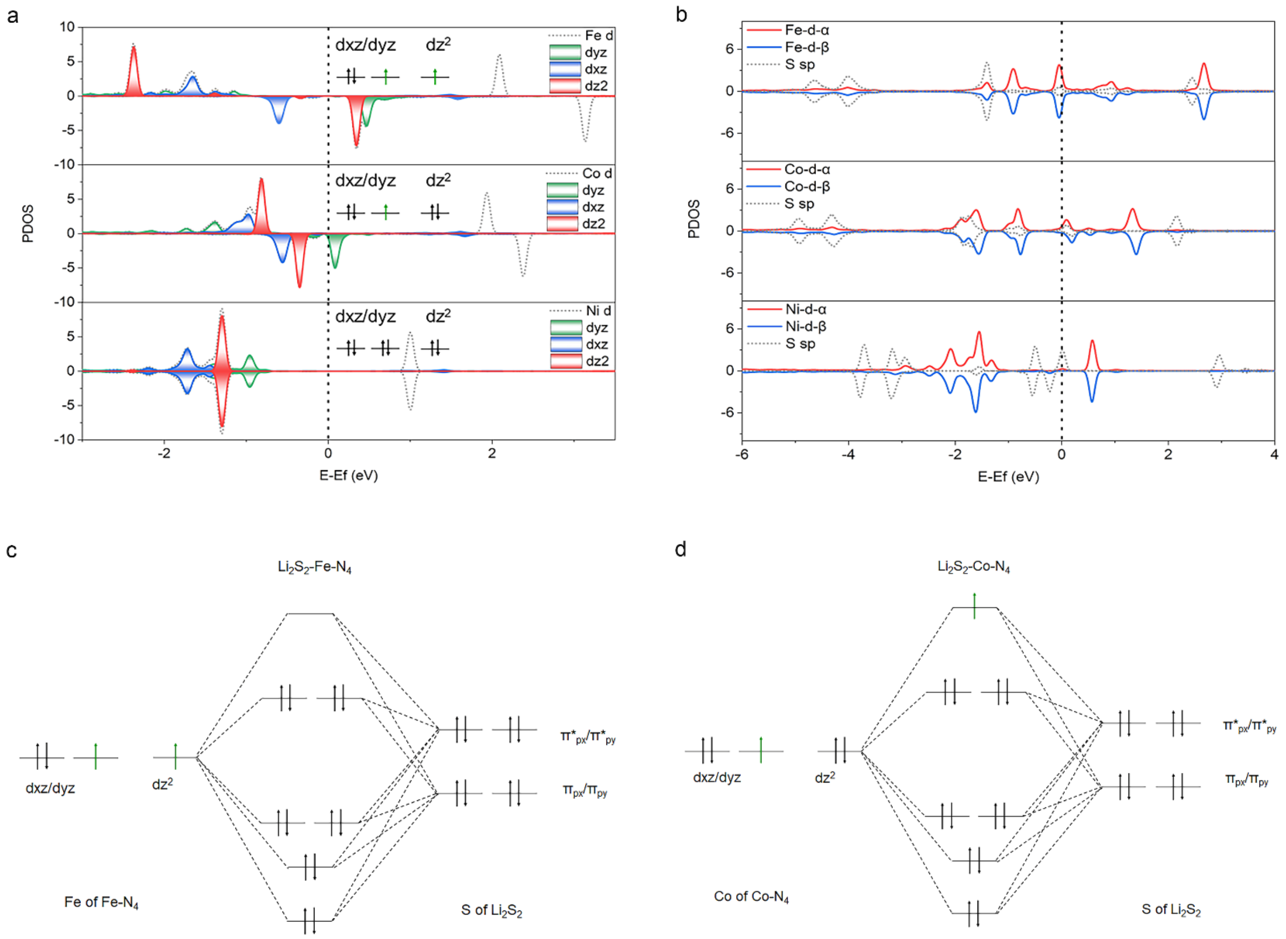

Figure S4. Electronic structure analysis of FEs-N_4 and Li_2S_2-FEs-N_4. The calculated PDOS of a) FEs-N_4 and b) Li_2S_2-FEs-N_4. The molecular orbital diagrams of c) Li_2S_2-Fe-N_4 and d) Li_2S_2-CoN_4 derive from b).

Through pDOS of the metal center in FEs-N_4 (Figure S4a), Fe-N_4 and Co-N_4 have arresting spin polarization phenomenon and the polarization degree satisfies the order of Fe > Co > Ni. Meantime, the inset shows the electrons filling in d orbitals, where Fe in Fe-N_4 possesses 2 unpaired electrons, exceeding corresponding Co (1 unpaired electron) and Ni (0 unpaired electron). This electron configuration is consistent with the magnetic moments (Fe: 1.91 μB, Co: 0.79 μB and Ni: 0 μB) and spin charge shapes (Fe: d_{z2} + d_{xz}/d_{yz}, Co: d_{xz}/d_{yz}) in Figure 1a.

When Li_2S_2 interacts with FEs-N_4 (Figure S4c-d), it can be seen that all electrons spin pairing for Li_2S_2-Fe-N_4, meantime, less antibonding orbitals are occupied in this system, suggesting a stable interaction. By contrast, Li_2S_2-Co-N_4 system still exists unpaired electron and occupies more antibonding orbitals than Li_2S_2-Fe-N_4. These results can be verified by the arrangement and spin

asymmetry of the orbit analysis of Li_2S_2-FEs-N_4 (Figure S4b).

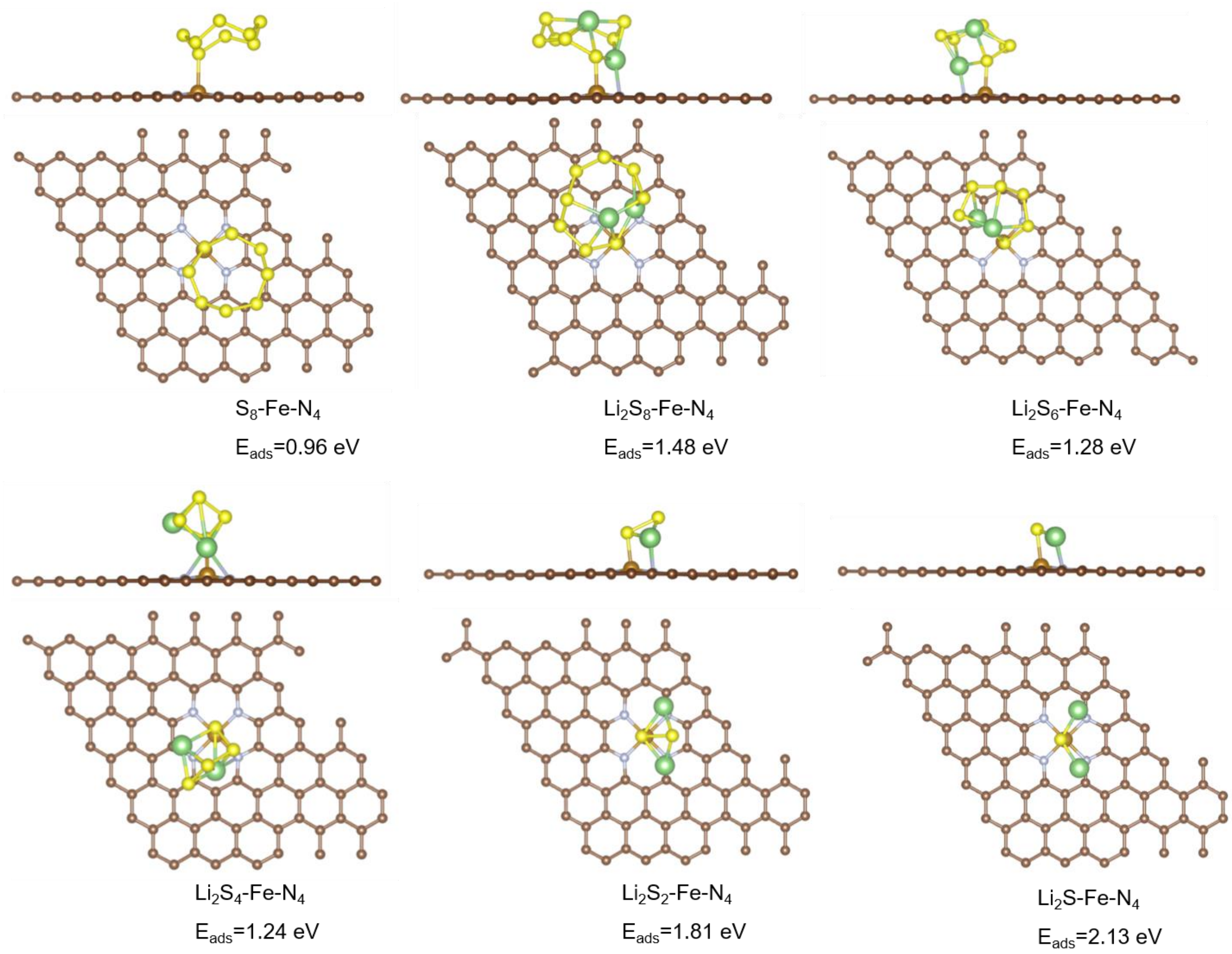

Figure S5. The optimized structures and adsorption energies of Li_2S_x ($1 \leq x \leq 8$) on the Fe-N_4.

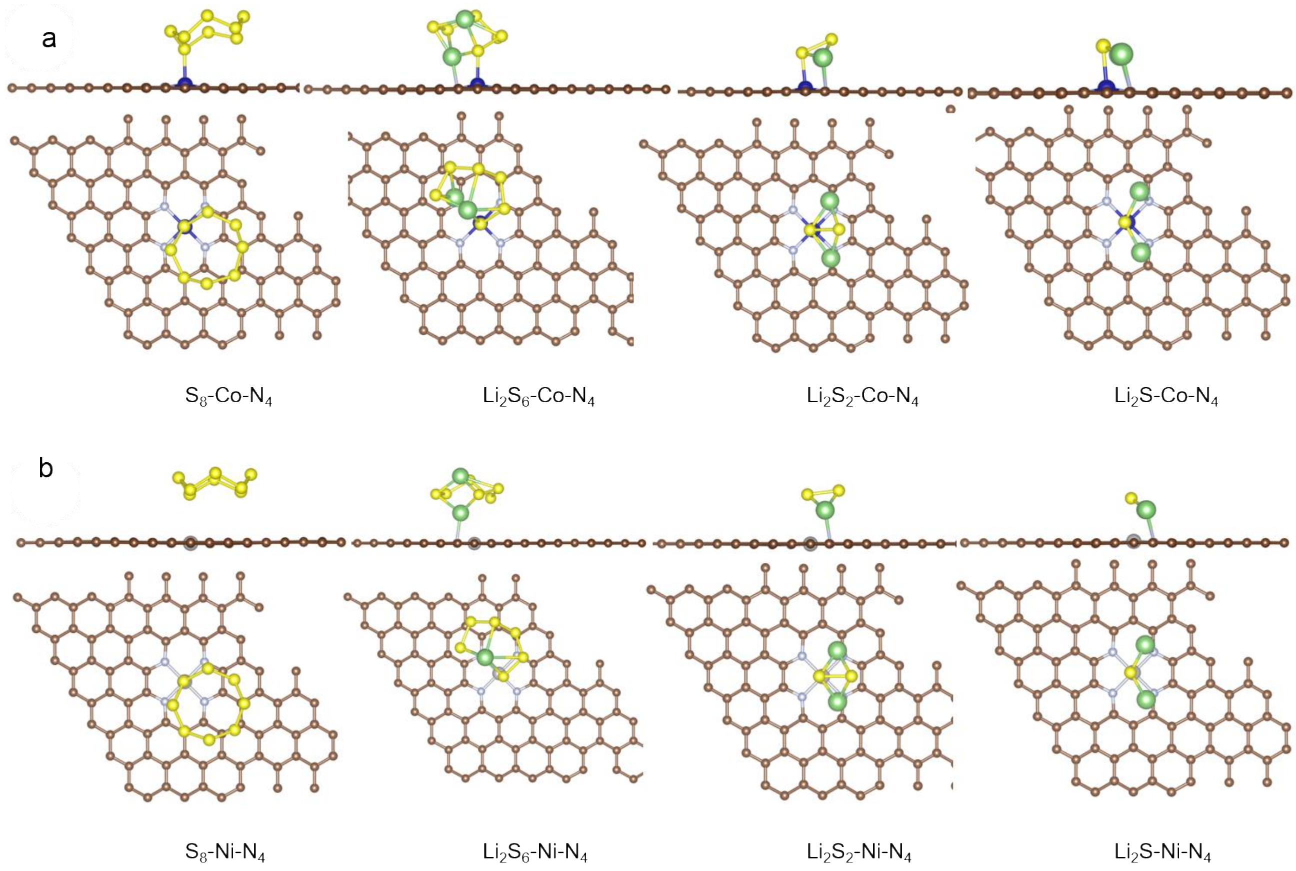

Figure S6. Optimized configurations of S_8, Li_2S_6, Li_2S_2, and Li_2S absorption on the Co-N_4 surface (a) and the Ni-N_4 surface (b).

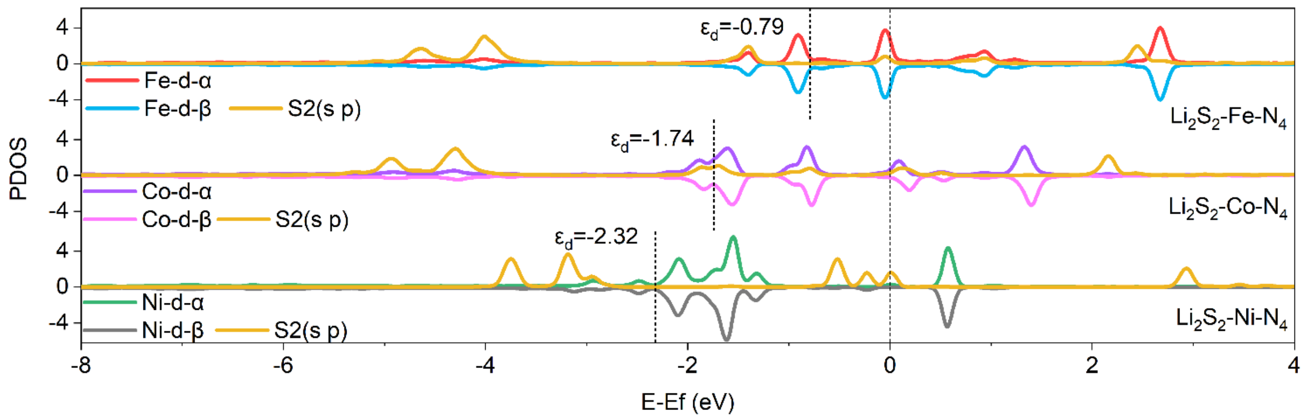

Figure S7. The calculated PDOS of Li_2S_2-Fe-N_4, Li_2S_2-Co-N_4, and Li_2S_2-Ni-N_4.

Table S1. The Bader charge (e^-) of S atom in Li_2S_2-FEs-N_4.

	Li_2S_2-Fe-N_4	Li_2S_2-Co-N_4	Li_2S_2-Ni-N_4
S1	0.73	0.70	0.70
S2	0.53	0.52	0.70
S1 - S2	0.20	0.18	0.00

Li_2S_2 is a symmetric molecule, so the Bader charge of S1 and S2 should be the same. However, after the interaction with FEs-N_4 substrate, the interaction between FEs and S will lead to the destruction of this symmetry of Li_2S_2 molecule, that is the Bader charge of S1 and S2 (the S that directly interacting with FEs) is no longer the same. This difference drives from the electron transfer of S2→FEs in Li_2S_2-FEs-N_4. Therefore, we acquire the electron transfer number S2→FEs via Bader charge difference value of S2 - S1 as shown in Table S1.

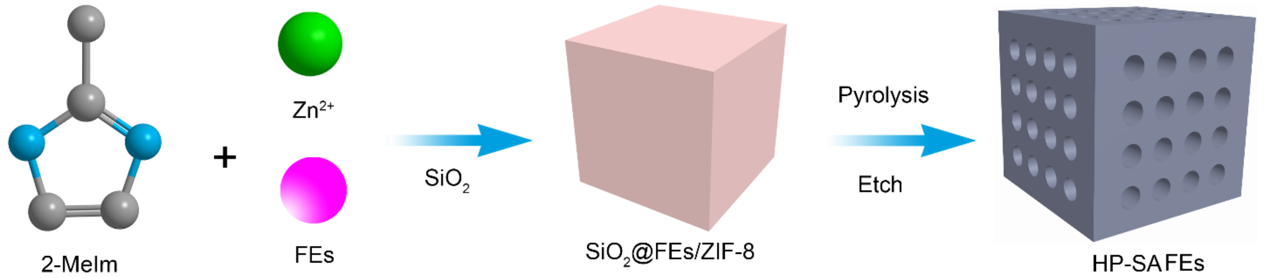

Figure S8. The preparation process of HP-SAFEs.

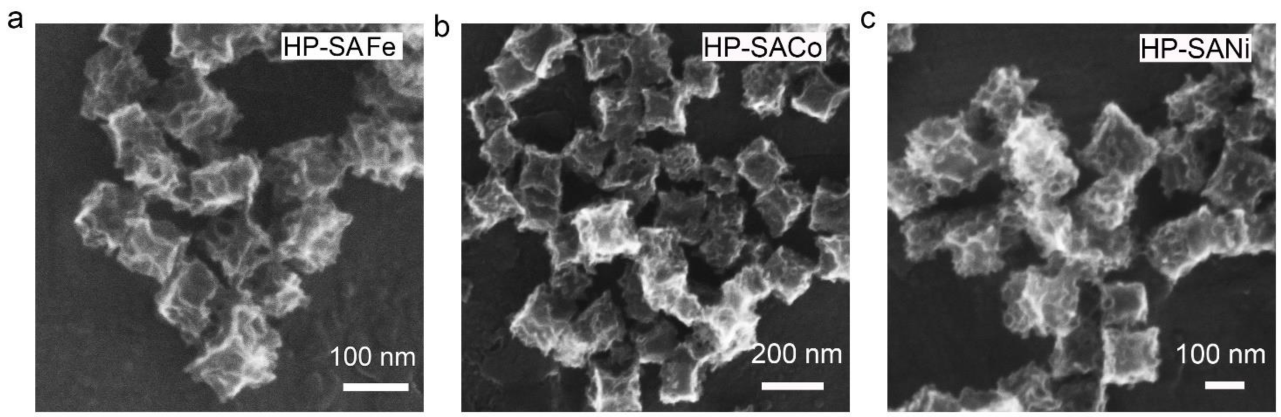

Figure S9. The SEM images of HP-SAFe (a), HP-SACo (b), and HP-SANi (c).

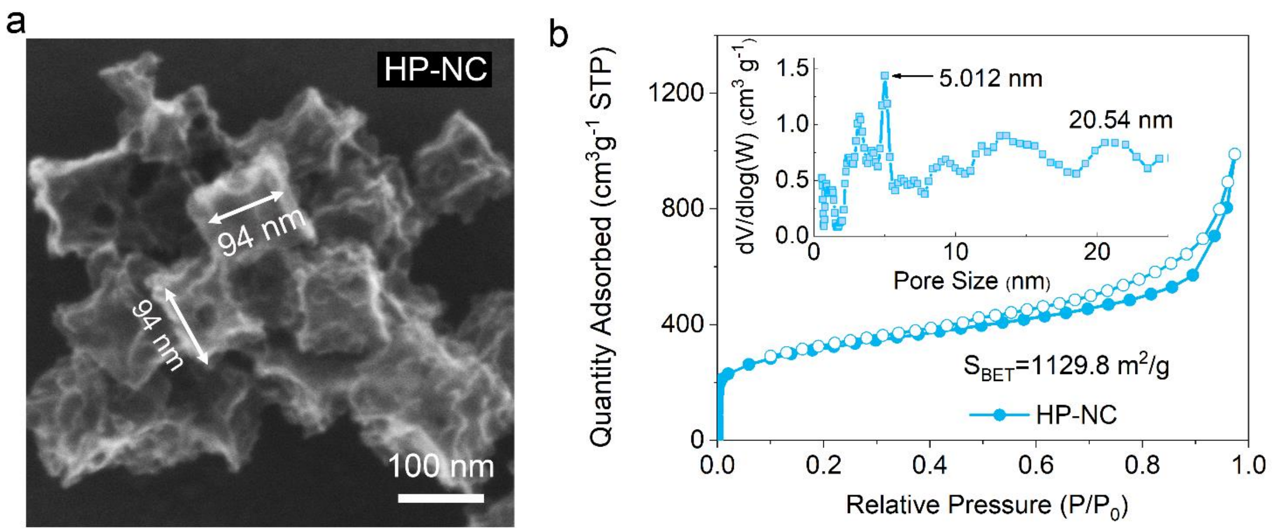

Figure S10. (a) The SEM images of HP-NC. (b) Nitrogen adsorption/desorption tests at 77 K for HP-NC.

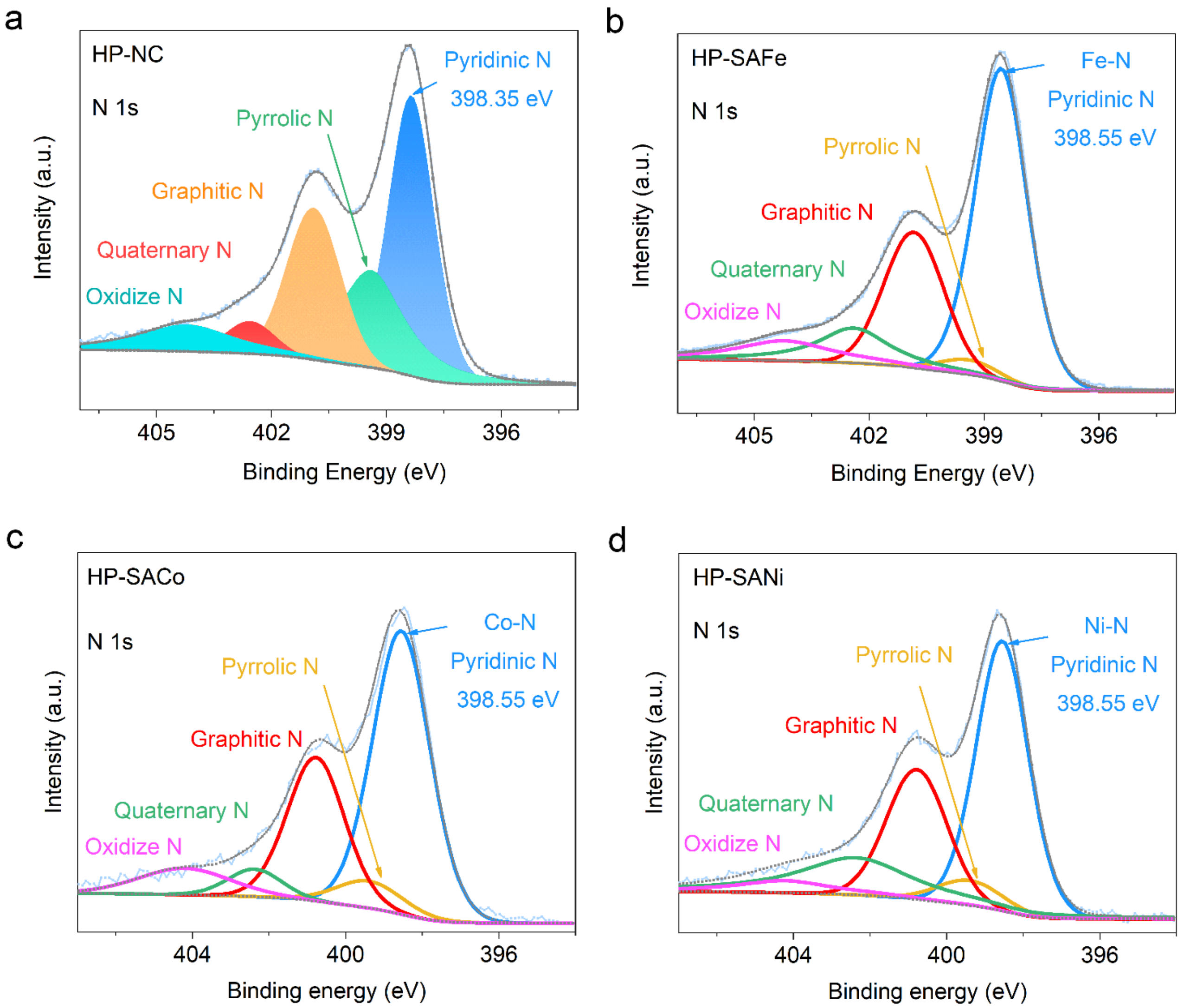

Figure S11. XPS spectra of N 1s for HP-NC (a), HP-SAFe (b), HP-SACo (c), and HP-SANi (d).

Table S2. Elemental atomic qualification results of C, N, O, Fe, Co, Ni (atom ratio %) in different electrode materials.

Samples	C (At %)	N (At %)	O (At %)	FEs (At %)
HP-SAFe	82.70	8.36	8.68	0.26
HP-SACo	79.03	12.54	8.01	0.42
HP-SANi	81.45	12.42	5.8	0.34

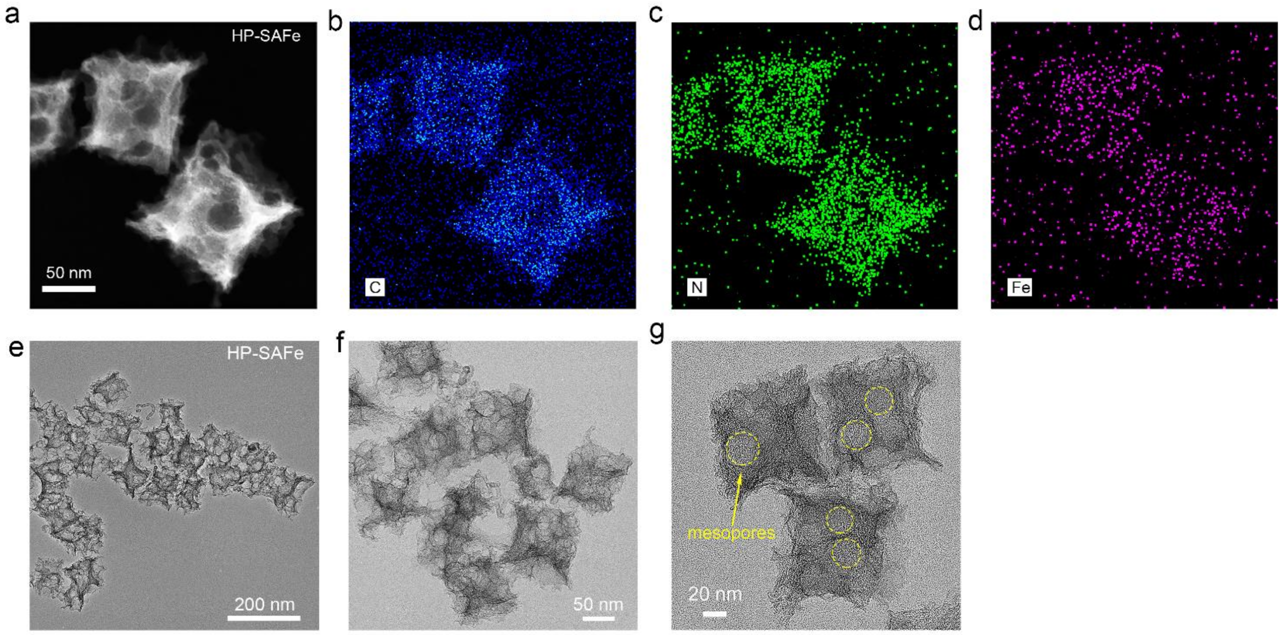

Figure S12. (a) The EDX elemental mappings of HP-SAFe for C (b), N (c), and Fe (d). (e-g) The TEM images of HP-SAFe.

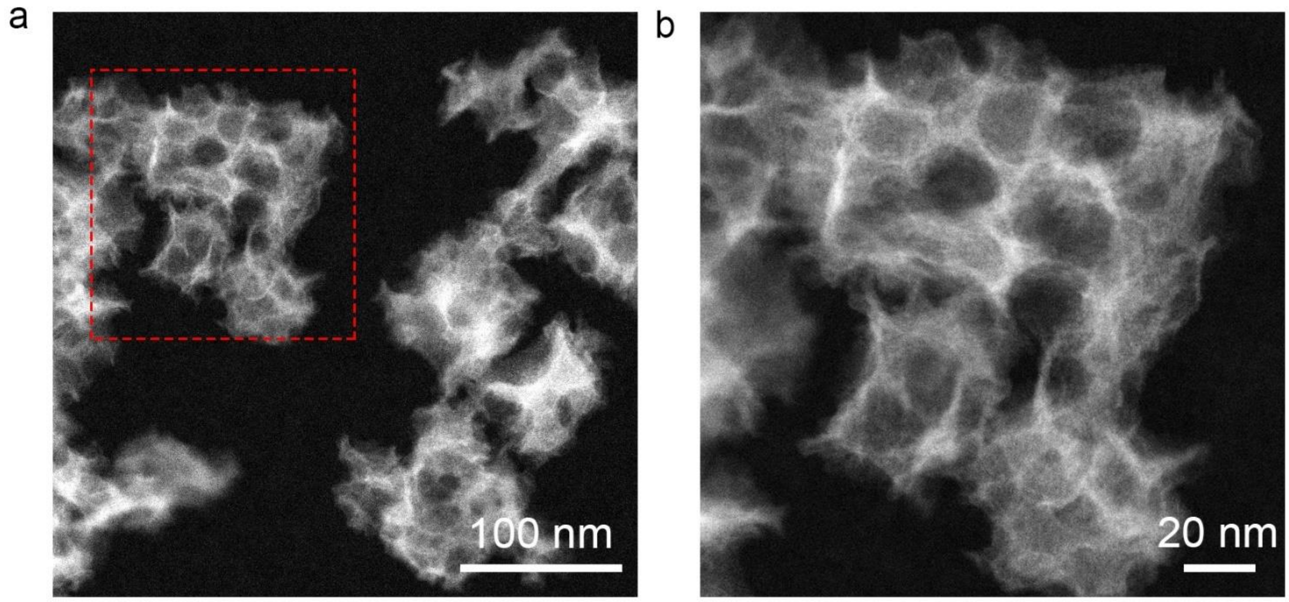

Figure S13. (a) HAADF-STEM image of HP-SAFe, and its magnification (b).

The aberration-corrected high-angle annular dark-field scanning transmission electron microscopy (HAADF-STEM) images of HP-SAFe reveal the cube morphologies and mesoporous architectures; meanwhile, no Fe nanoparticles are observed among the carbon frameworks.

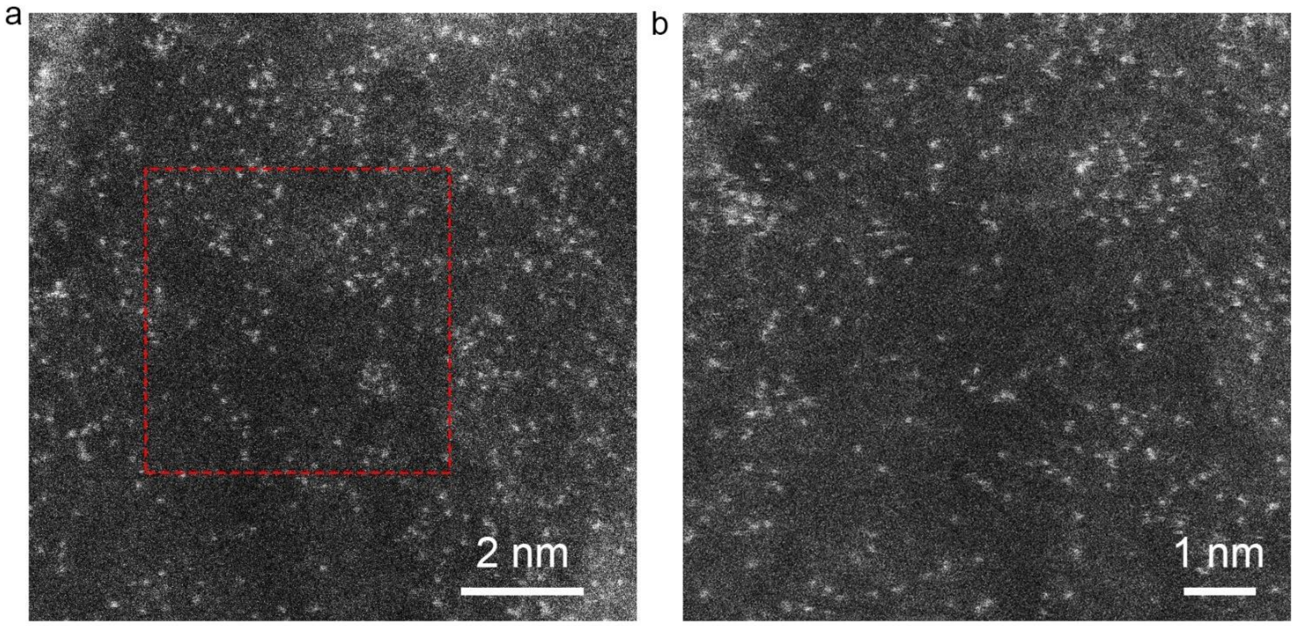

Figure S14. (a-b) AC-STEM images of HP-SAFe. Fe single atoms were the bright spots.

Table S3. EXAFS fitting parameters at the Fe K-edge for various samples (S_0^2=0.776)

Sample	Shell	N^a	R(Å)^b	σ^2(Å^2)^c	ΔE_0(eV)^d	R factor
Fe foil	Fe-Fe	8	2.47	0.0050	6.9	0.0023
	Fe-Fe	6	2.85	0.0064		
Fe_2O_3	Fe-O	6.0	2.04	0.0097	0.6	0.0009
	Fe-Fe	6.0	2.97	0.0075		
	Fe-Fe	4.1	3.38	0.0075		
	Fe-Fe	5.9	3.71	0.0075		
FePc	Fe-N	4.0	2.01	0.0084	1.0	0.0001
	Fe-C	7.9	3.02	0.0049		
	Fe-C	5.0	3.32	0.0049		
HP-SAFe	Fe-N	4.1	1.99	0.0055	-4.4	0.0004
Co foil	Co-Co	12	2.49	0.0050	7.3	0.00004
HP-SACo	Co-N	4.0	1.75	0.0050	7.2	0.0002
Ni foil	Ni-Ni	12	2.48	0.0059	6.4	0.00017
HP-SANi	Ni-N	4.1	1.71	0.0080	6.2	0.0004

$^a N$: coordination numbers; $^b R$: bond distance; $^c\sigma^2$: Debye-Waller factors; ^{d} ΔE_0: the inner potential correction. R factor: goodness of fit. S_0^2 was set to 0.776, according to the experimental EXAFS fit of Fe foil by fixing CN as the known crystallographic value.

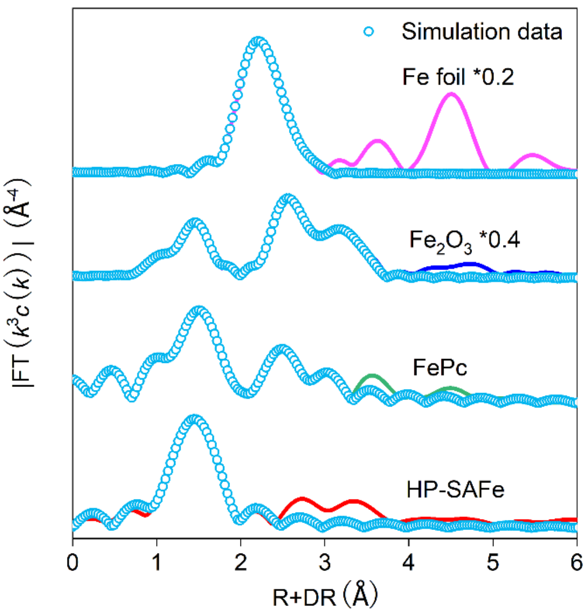

Figure S15. EXAFS spectra and analysis of Fe, Fe_2O_3, FePc, and HP-SAFe at Fe K-edges.

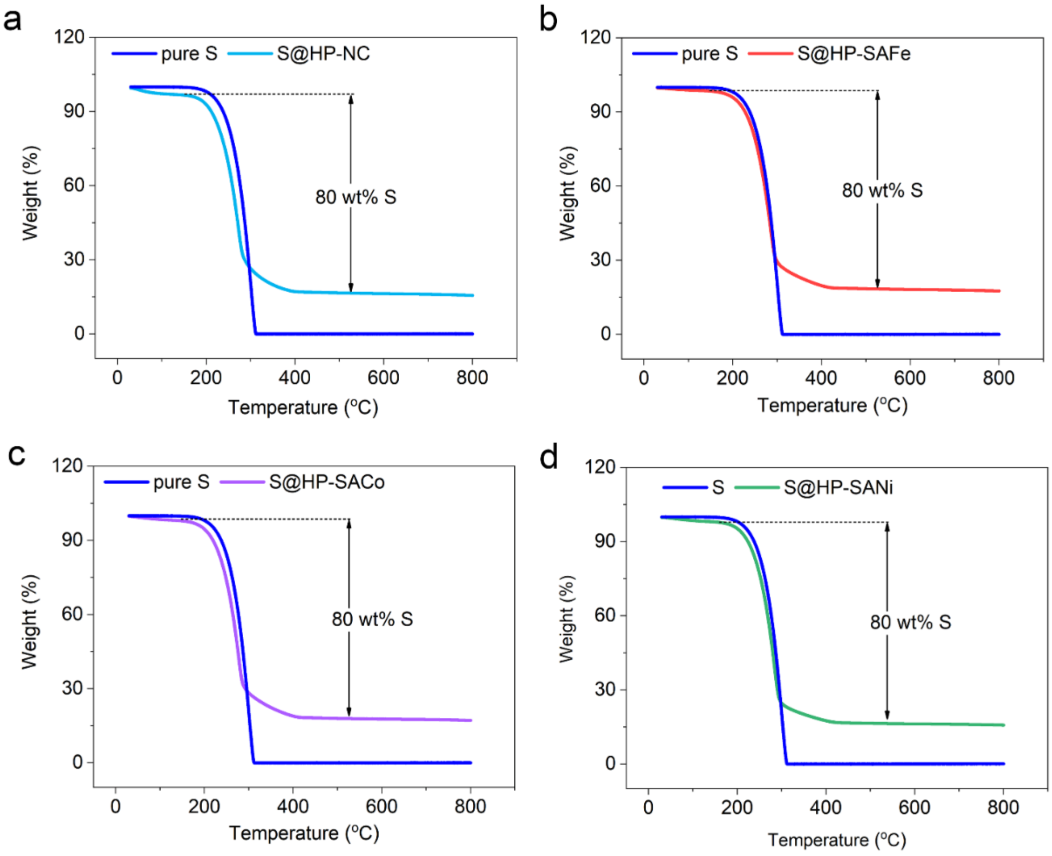

Figure S16. The TGA curves of S@HP-NC (a), S@HP-SAFe (b), S@HP-SACo (c), and S@HP-SANi (d).

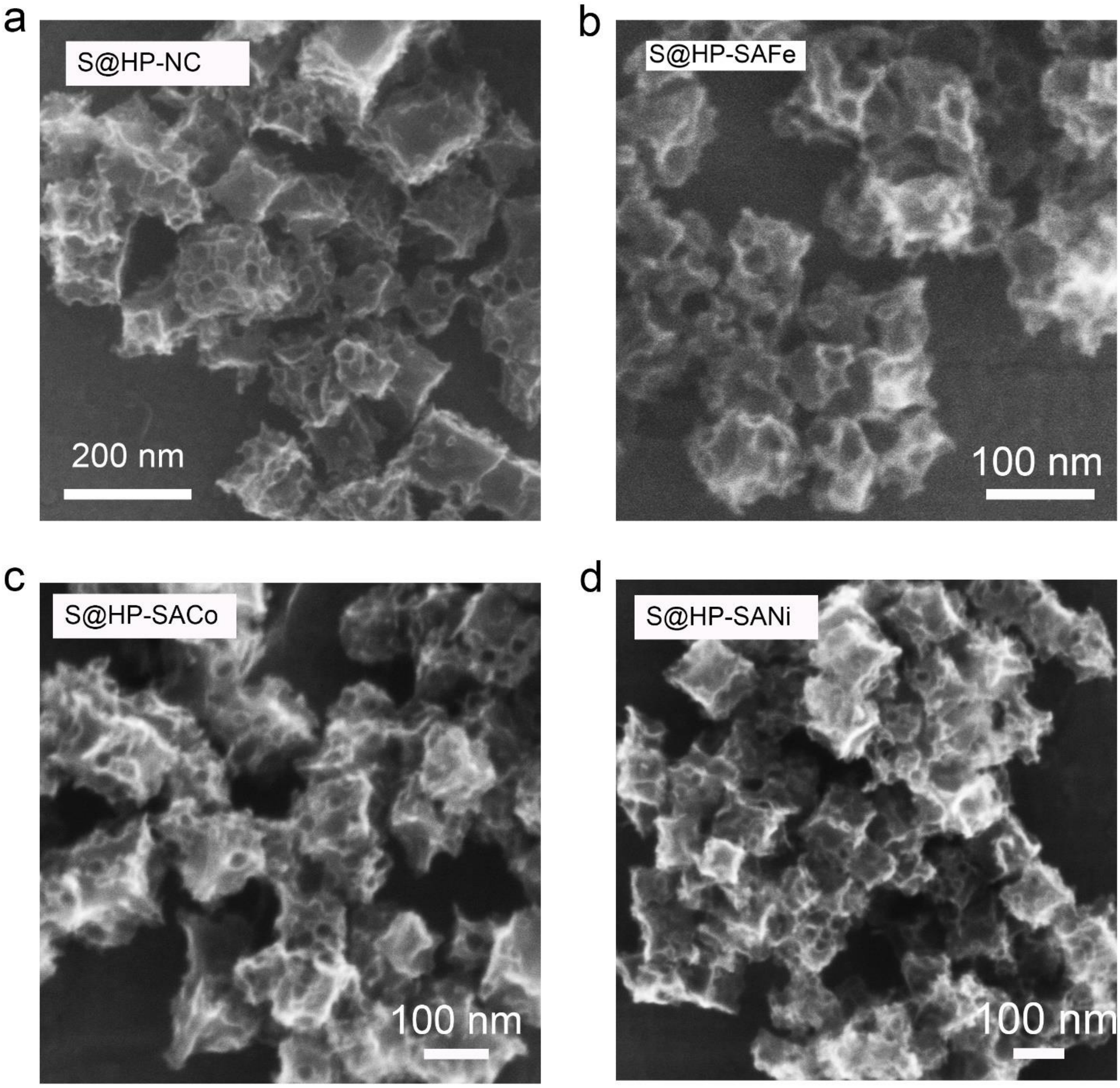

Figure S17. The SEM images of S@HP-NC (a), S@HP-SAFe (b), S@HP-SACo (c), and S@HP-SANi (d).

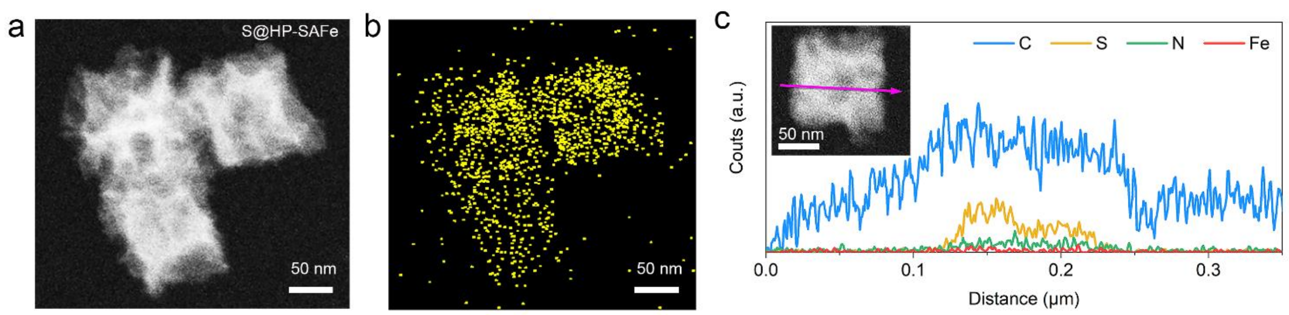

Figure S18. (a) The HAADF-STEM image of S@HP-SAFe and (b) corresponding EDX elemental mapping of S. (c) Elemental linear distribution images of S@HP-SAFe composite.

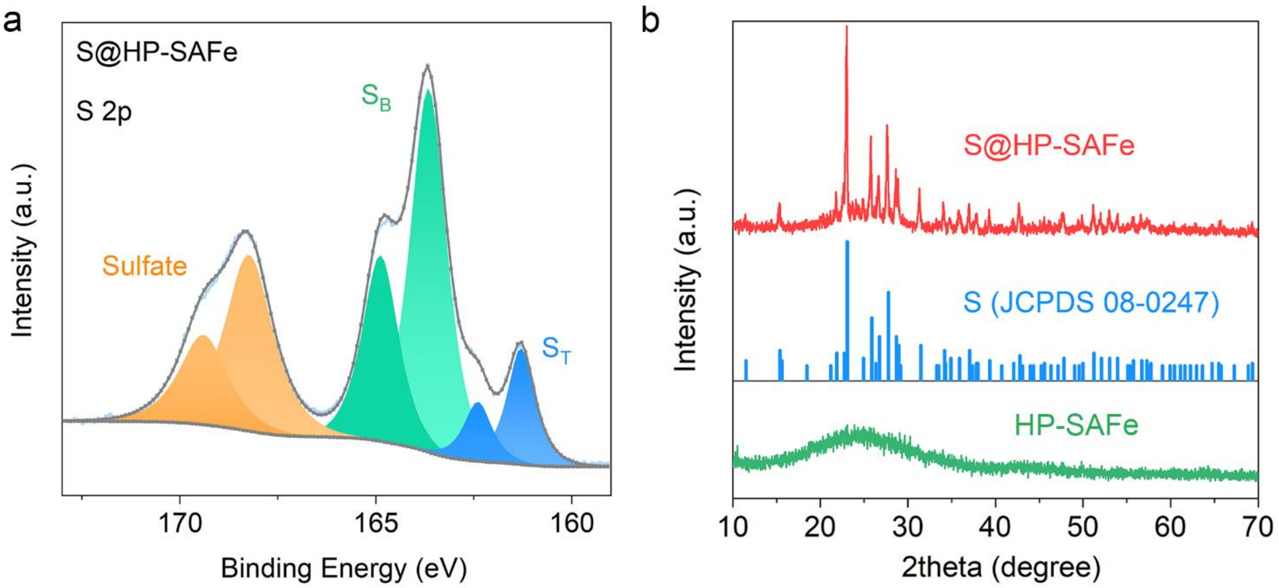

Figure S19. (a) High-resolution XPS S2p analysis of S@HP-SAFe. (b) XRD analysis of S@HP-SAFe.

Figure S17a show the high-resolution XPS spectra of the S 2p spectrum of S@HP-SAFe, the peaks at 161.3 and 162.4 eV are attributed to the terminal sulfur (S_T), and the peaks at 163.6 and 164.8 eV result from the bridging sulfur (S_B). The detected signals at 168.2 and 169.4 eV can be assigned to the sulfate.

The XRD patterns of S@HP-SAFe agree well with sulfur (JCPDS 08-0247). Meantime, one can see that there are no characteristic peaks of Fe crystals in the XRD pattern of HP-SAFe, demonstrating the poor crystallinity.

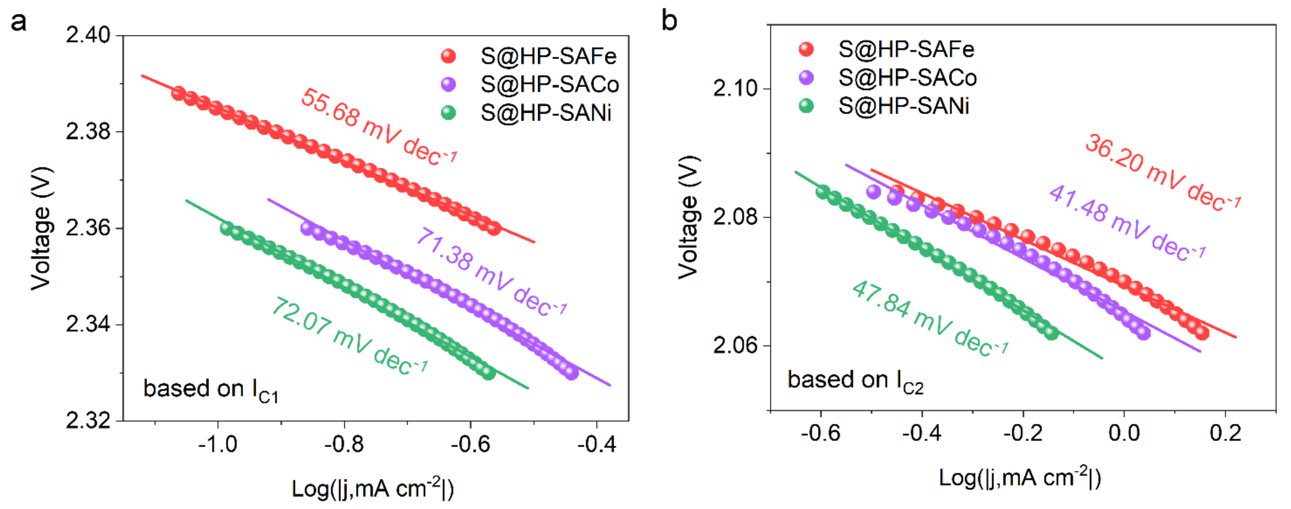

Figure S20. The Tafel plots of HP-SAFe, HP-SACo, and HP-SANi are based on the I_{C1} peak (a) and the I_{C2} peak (b).

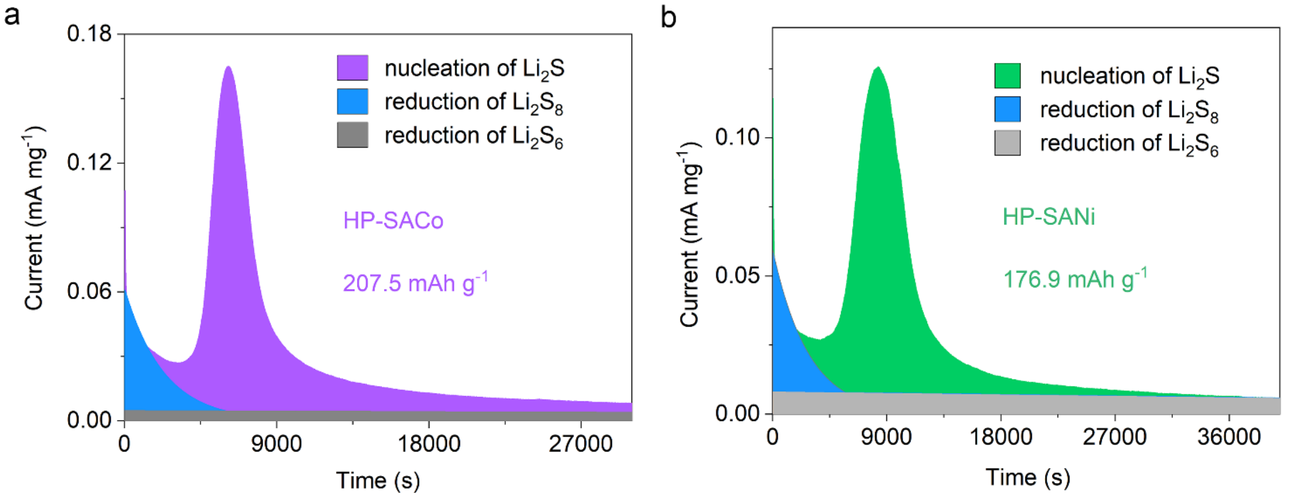

Figure S21. Potentiostatic tests for HP-SACo (a) and HP-SANi (b).

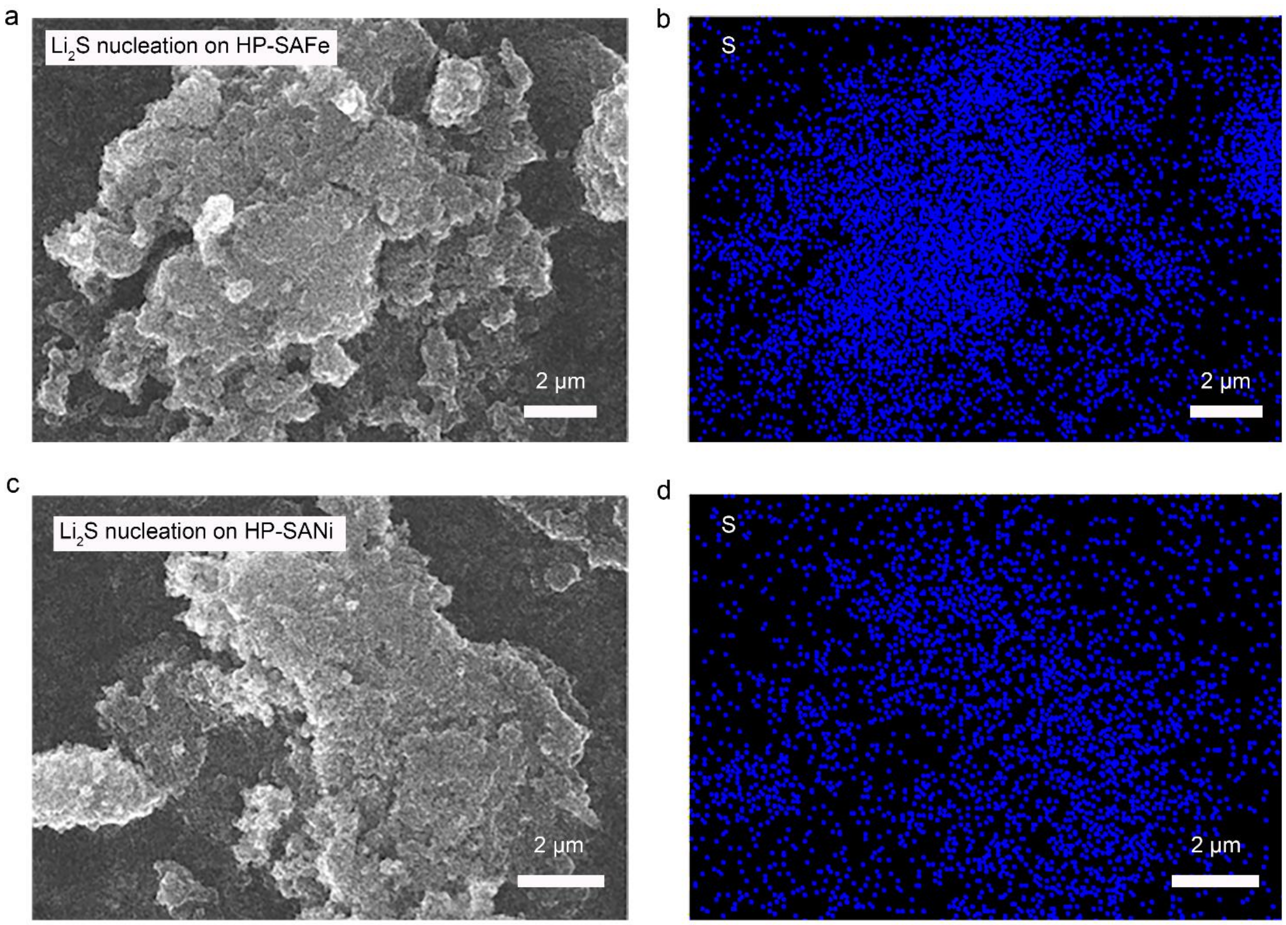

Figure S22. (a) SEM and EDS images of electrodeposition tests on the HP-SAFe and (b) HP-SANi.

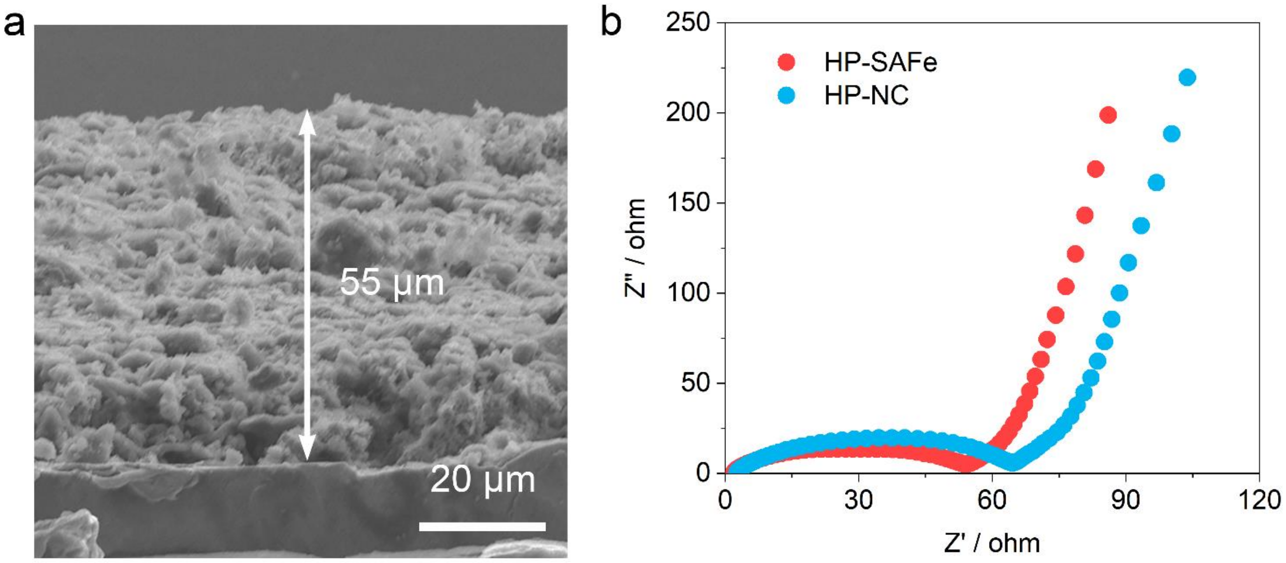

Figure S23. (a) The thickness of the S@HP-SAFe electrode. (b) Nyquist plot of the S@HP-SAFe and S@HP-NC cathodes.

The thickness of the S@HP-SAFe electrode is confirmed by SEM to be around 55 μm, meantime, Figure S22b presents the electrochemical impedance spectra (EIS) at open-circuit voltage between 100 kHz and 10 mHz, where a shorter semicircle diameter for the S@HP-SAFe than S@HP-NC cathodes indicates the fast charge transfer.

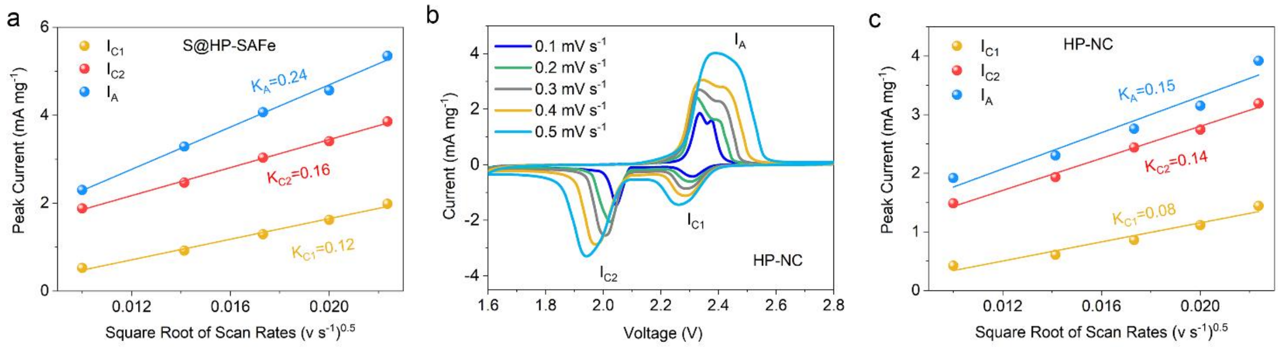

Figure S24. (a) Plots of peak currents (i_p) with the square root of the scan rate ($v^{1/2}$) for the cathodic reduction processes (I_{C1}, I_{C2}) and anodic oxidation process (I_A) in Li-S batteries with S@HP-SAFe. (b) CV tests of HP-NC at different scan rates from 0.1 to 0.5 mV s^{-1}. (c) i_p with the $v^{1/2}$ for the I_{C1}, I_{C2}, and I_A in Li-S batteries with S@HP-NC.

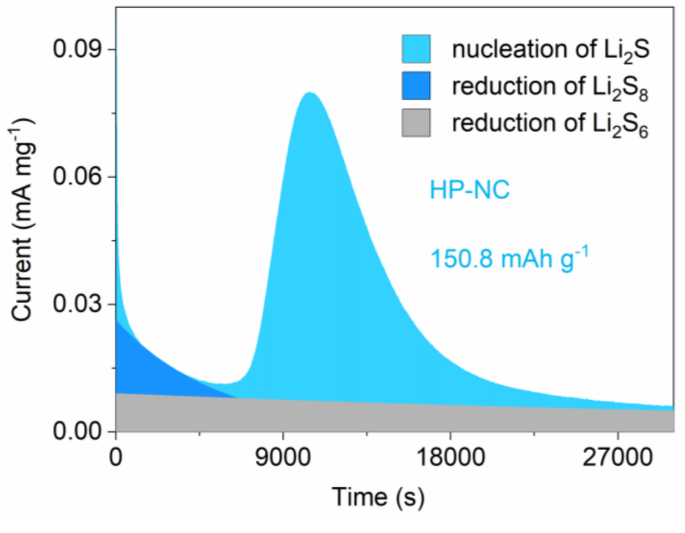

Figure S25. Potentiostatic discharge curves with Li_2S_8 catholyte for HP-NC.

The kinetics of the liquid-solid reaction from polysulfides to Li_2S was investigated with a potentiostatic method at 2.05 V after the first galvanostatic discharging process at a current of 0.112 mA. The integral areas of the current peaks corresponding to the nucleation of Li_2S were calculated

based on Faraday's law. The value for the HP-NC is 150.8 mAh g^{-1}, which is much lower than that of HP-SAFe (226.1 mAh g^{-1}).

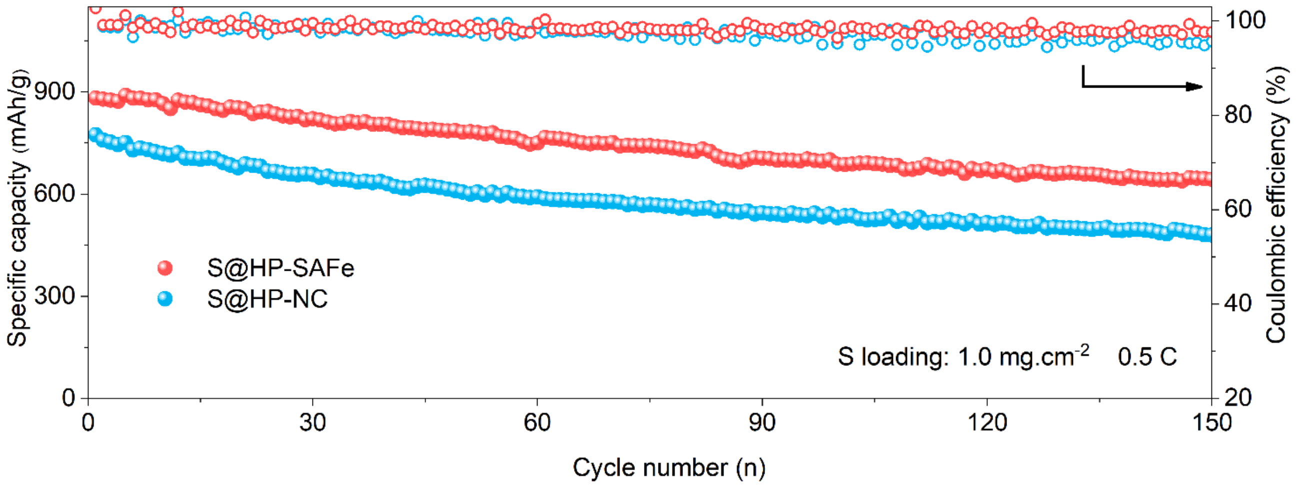

Figure S26. Cycling stability of HP-NC and HP-SAFe-based sulfur cathodes at 0.5 C with the sulfur loading of 1 mg cm^{-2}.

Table S4. Comparison of polysulfide catalysts reported to date for lithium-sulfur batteries.

Catalysts	S loading (mg cm^{-2})	Initial capacity (mAh g^{-1})	Reversible capacity (mAh g^{-1})	Capacity decay per cycle (%)	Rate/Cycles	Ref.
$Ni_{0.1}Zn_{0.1}Co_{0.8}Se_2$-S	1	679.47	502.81	0.065	1 C/400	[7]
RG@CoS@C	2	927.9	629.2	0.08	2 C/420	[8]
VN@NG	1	884	509	0.075	2 C/500	[9]
TS-Ti_3C_2/CNT	1.2	1010	610	0.08	1 C/500	[10]
DPDSe	1.2	1056	720	0.091	0.5 C/350	[11]
Co_3Se_2	1.5	1156	752	0.17	0.5 C/200	[12]
TiS_2@NSC	2.5	1019	695	0.16	1 C/200	[13]
C-Co/TiO_2	1.5	600	384	0.12	2 C/300	[14]
Li_2S@NC:SAFe	2	1052	790	0.12	1 C/200	[15]
Fe-N-C	1.3	1027	427	0.2	0.1 C/300	[16]

SC-Co	1.2	1095	837	0.086	0.5 C/300	[17]
Co-N-C	2.8	1161	850	0.1	0.5 C/300	[18]
**HP-SAFe**	**1**	**688**	**451**	**0.087**	**2 C/400**	**This work**

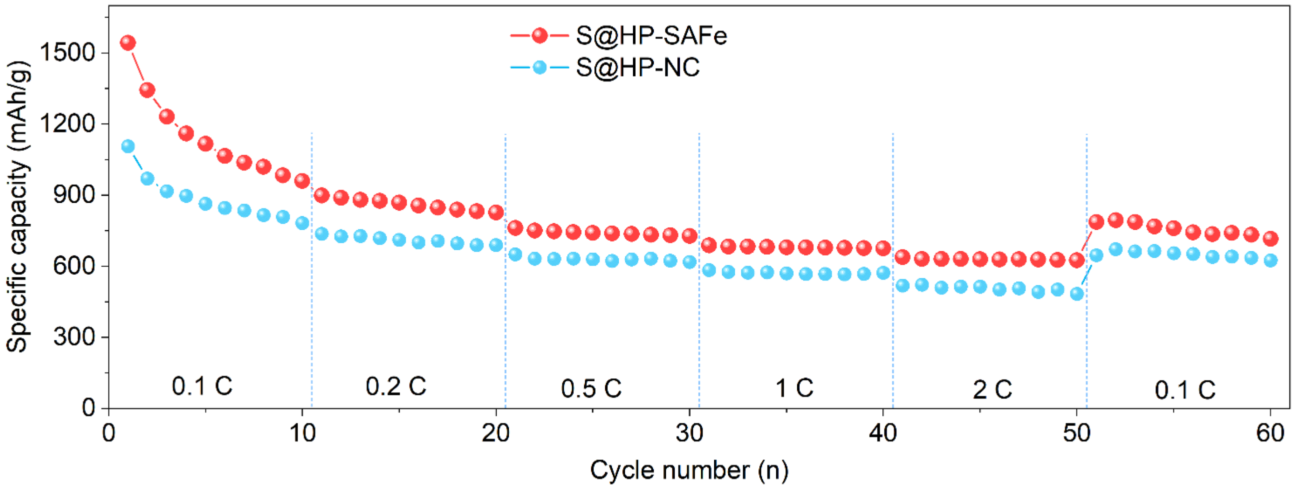

Figure S27. The rate capability of HP-NC and HP-SAFe-based sulfur cathodes from 0.1 C to 2.0 C.

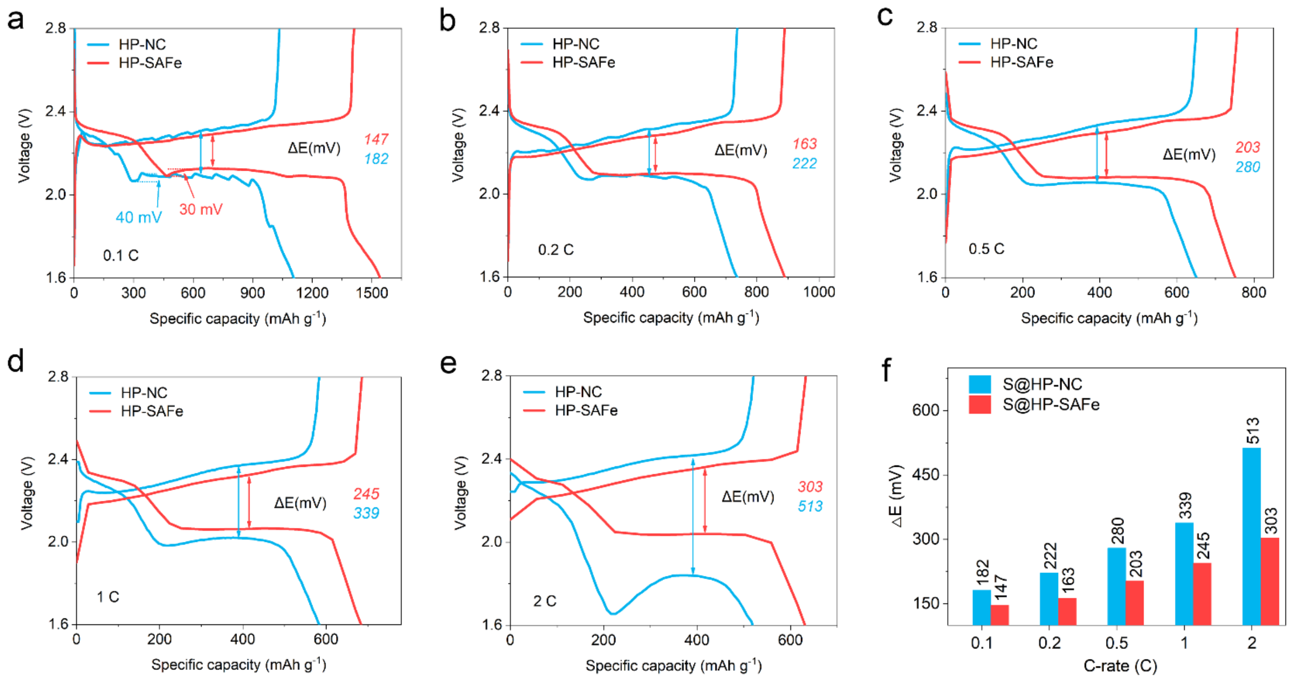

Figure S28. Charge-discharge curves of S@HP-SAFe, S@HP-NC, and S@NC at 0.1 C (a), 0.2 C (b), 0.5 C (c), 1 C (d), 2 C (e). (f) Potential difference between the anodic and cathodic sweep in HP-NC and HP-SAFe-based sulfur cathodes at different C rates.

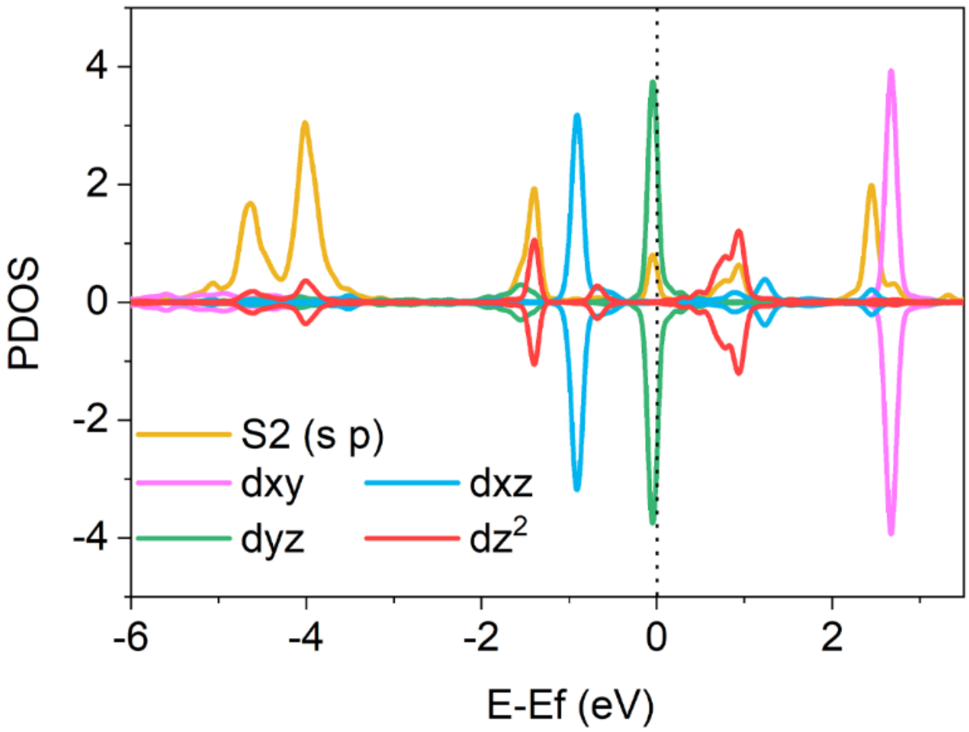

Figure S29. The calculated PDOS of Li_2S_2-Fe-N_4.

References:

[1] a) G. Kresse, J. Furthmüller, *Phy. Rev. B.* **1996**, *54*, 11169-11186; b) G. Kresse, J. Furthmüller, *Comp. Mater. Sci.* **1996**, *6*, 15-50; cG. Kresse, J. Hafner, *Phys. Rev. B.* **1994**, *49*, 14251-14269.

[2] P. E. Blöchl, *Phys. Rev. B.* **1994**, *50*, 17953-17979.

[3] J. P. Perdew, J. A. Chevary, S. H. Vosko, K. A. Jackson, M. R. Pederson, D. J. Singh, C. Fiolhais, *Phys. Rev. B.* **1992**, *46*, 6671-6687.

[4] a) J. Klimeš, D. R. Bowler, A. Michaelides, *J. Phys.: Condens. Matter.* **2009**, *22*, 022201; b) J. Klimeš, D. R. Bowler, A. Michaelides, *Phy. Rev. B.* **2011**, *83*, 195131.

[5] a) W. Tang, E. Sanville, G. Henkelman, *J. Phys.: Condens. Matter.* **2009**, *21*, 084204; b) R. F. W. Bader, *Chem. Rev.* **1991**, *91*, 893-928.

[6] a) J. Yu, J. Xiao, A. Li, Z. Yang, L. Zeng, Q. Zhang, Y. Zhu, L. Guo, *Angew. Chem. Int. Ed.* **2020**, *59*, 13071-13078; b) H. Park, H. S. Koh, D. J. Siegel, *J. Phys. Chem. C.* **2015**, *119*, 4675-4683.

[7] Z. Zeng, L. Y. Gan, H. Bin Yang, X. Su, J. Gao, W. Liu, H. Matsumoto, J. Gong, J. Zhang, W. Cai, Z. Zhang, Y. Yan, B. Liu, P. Chen, *Nat. Commun.* **2021**, *12*, 4088.

[8] J. Guo, H. Jiang, X. Li, Z. Chu, W. Zheng, Y. Dai, X. Jiang, X. Wu, G. He, *Energy Storage Mater.* **2021**, *40*, 358-367.

[9] G. Yang, J. Zhu, P. Yuan, Y. Hu, G. Qu, B.-A. Lu, X. Xue, H. Yin, W. Cheng, J. Cheng, W. Xu, J. Li, J. Hu, S. Mu, J.-N. Zhang, *Nat. Commun.* **2021**, *12*, 1734.

[10] X. Wang, D. Luo, J. Wang, Z. Sun, G. Cui, Y. Chen, T. Wang, L. Zheng, Y. Zhao, L. Shui, G. Zhou, K. Kempa, Y. Zhang, Z. Chen, *Angew. Chem. Int. Ed.* **2021**, *60*, 2371-2378.

[11] M. Zhao, X. Chen, X.-Y. Li, B.-Q. Li, J.-Q. Huang, *Adv. Mater.* **2021**, *33*, 2007298.

[12] D. Cai, B. Liu, D. Zhu, D. Chen, M. Lu, J. Cao, Y. Wang, W. Huang, Y. Shao, H. Tu, W. Han, *Adv. Energy Mater.* **2020**,

10, 1904273.

[13] X. Huang, J. Tang, B. Luo, R. Knibbe, T. Lin, H. Hu, M. Rana, Y. Hu, X. Zhu, Q. Gu, D. Wang, L. Wang, *Adv. Energy Mater.* **2019**, *9*, 1901872.

[14] R. Liu, Z. Liu, W. Liu, Y. Liu, X. Lin, Y. Li, P. Li, Z. Huang, X. Feng, L. Yu, D. Wang, Y. Ma, W. Huang, *Small.* **2019**, *15*, 1804533.

[15] J. Wang, L. Jia, J. Zhong, Q. Xiao, C. Wang, K. Zang, H. Liu, H. Zheng, J. Luo, J. Yang, H. Fan, W. Duan, Y. Wu, H. Lin, Y. Zhang, *Energy Storage Mater.* **2019**, *18*, 246-252.

[16] Z. Liu, L. Zhou, Q. Ge, R. Chen, M. Ni, W. Utetiwabo, X. Zhang, W. Yang, *ACS Appl. Mater. Interfaces.* **2018**, *10*, 19311-19317.

[17] J. Xie, B.-Q. Li, H.-J. Peng, Y.-W. Song, M. Zhao, X. Chen, Q. Zhang, J.-Q. Huang, *Adv. Mater.* **2019**, *31*, 1903813.

[18] B.-Q. Li, L. Kong, C.-X. Zhao, Q. Jin, X. Chen, H.-J. Peng, J.-L. Qin, J.-X. Chen, H. Yuan, Q. Zhang, J.-Q. Huang, *InfoMat.* **2019**, *1*, 533-541.
